# Pseudo‐MRI Engine for MRI‐Free Electromagnetic Source Imaging

**DOI:** 10.1002/hbm.70148

**Published:** 2025-02-04

**Authors:** Amit Jaiswal, Jukka Nenonen, Lauri Parkkonen

**Affiliations:** ^1^ Department of Neuroscience and Biomedical Engineering School of Science, Aalto University Espoo Finland; ^2^ Megin Oy Espoo Finland

**Keywords:** digitization, EEG, forward modeling, MEG, source estimation, template MRI, thin plate spline, warping

## Abstract

Structural head MRIs are a crucial ingredient in MEG/EEG source imaging; they are used to define a realistically shaped volume conductor model, constrain the source space, and visualize the source estimates. However, individual MRIs are not always available, or they may be of insufficient quality for segmentation, leading to the use of a generic template MRI, matched MRI, or the application of a spherical conductor model. Such approaches deviate the model geometry from the true head structure and limit the accuracy of the forward solution. Here, we implemented an easy‐to‐use tool, *pseudo‐MRI engine*, which utilizes the head‐shape digitization acquired during a MEG/EEG measurement for warping an MRI template to fit the subject's head. To this end, the algorithm first removes outlier digitization points, densifies the point cloud by interpolation if needed, and finally warps the template MRI and its segmented surfaces to the individual head shape using the thin‐plate‐spline method. To validate the approach, we compared the geometry of segmented head surfaces, cortical surfaces, and canonical brain regions in the real and pseudo‐MRIs of 25 subjects. We also tested the MEG source reconstruction accuracy with pseudo‐MRIs against that obtained with the real MRIs from individual subjects with simulated and real MEG data. We found that the pseudo‐MRI enables comparable source localization accuracy to the one obtained with the subject's real MRI. The study indicates that pseudo‐MRI can replace the need for individual MRI scans in MEG/EEG source imaging for applications that do not require subcentimeter spatial accuracy.

## Graphical abstract

A tool, pseudo‐MRI engine, was implemented to generate subject‐specific template MRIs based on scalp digitization that can readily be used for MEG/EEG source imaging. The geometrical and source‐imaging similarity with real MRI proves that the method can be an alternative to individual MRIs in various MEG/EEG applications.

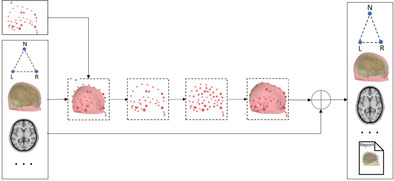

**Summary**

- Structural magnetic resonance imaging (MRI) of the subject's head is crucial for spatial accuracy in electromagnetic source imaging, but such an MRI may not always be available.
- We implemented an easy‐to‐use tool, *pseudo‐MRI engine*, that generates a subject‐specific template MRI based on the head shape. Such a pseudo‐MRI can readily be used with several software packages for MEG/EEG source imaging.
- Our engine inputs the digitized head shape, removes outlier points, increases the point density, and applies adaptive regularization to warp a template MRI to the head shape precisely. The engine provides a set of volumetric MRI segments along with head and cortical surfaces.
- To validate the approach, we compared real and pseudo‐MRI surfaces in 25 healthy adults. We also compared source‐imaging accuracy for simulated and real MEG responses.
- Our results show that a *pseudo‐MRI* can be a viable alternative to an individual MRI when subcentimeter spatial accuracy in source imaging is not required.

## 1 Introduction

Structural (anatomical) magnetic resonance imagings (MRIs) are an integral part of most MEG/EEG source‐imaging workflows. T1‐weighted MRIs and automatic MRI segmentation software packages are used to generate head‐surface meshes, cortical surface meshes, and atlas‐based parcellations of the cortical surface (Baillet et al. 2001; Dale et al. 2000; Gramfort et al. 2013; Gross et al. 2013; Ilmoniemi 2020; Liu et al. 2006). These derivatives are typically used to define a subject‐specific volume conductor model and cortically constrained or volumetric source space to compute the forward solution. MRIs are also employed for visualizing the source estimates. For these purposes, it is important to use subject‐specific MRIs, since the structural variability between individual brains is known to be substantial, and even an individual brain varies significantly across age (Fjell et al. 2015). Therefore, a T1‐weighted MRI is a useful representation of the MEG/EEG‐relevant structural boundaries of the brain and head, and is highly recommended in MEG/EEG source imaging (Henson et al. 2009; Mosher et al. 1999). Such an MRI should be recent enough to reflect the current brain anatomy such that there are no significant structural changes in the brain between the MRI scan and MEG/EEG measurement. Usually, an MRI with 1‐mm isotropic voxels covering the whole head at a contrast sufficient for segmentation and reconstruction of the head and cortical surfaces, and other relevant tissue boundaries. The shapes of the conductor‐model surfaces and cortical mantle are often checked visually to ensure the reconstruction quality.

Obtaining an MRI, however, is an extra step with an associated cost, and it usually requires that the subject visits the MRI facility separately from the MEG/EEG measurement session. Also, there can be obstacles that prohibit the use of an individual MRI; (i) structural MRI from the individual cannot be acquired due to practical constraints such as specific disabilities, MRI‐incompatible implants, or assistive devices, (ii) MRI scans for research purposes may be prohibited due to ethical constraints, for example in young children (Barkovich et al. 2019), (iii) the quality (contrast) of the MRI is not adequate for correct segmentation, or (iv) the field‐of‐view of an existing MRI does not include the whole head, which may prevent reconstructing the required surfaces for co‐registration and for defining the volume conductor model. Sometimes, contraindications for MRI also preclude MEG; however, advances in MEG noise suppression methods such as tSSS (Nenonen et al. 2012; Taulu et al. 2005; Taulu and Simola 2006) have expanded the application of MEG even in patients with medical implants (Bahners et al. 2024; Hnazaee et al. 2023; Mosher and Funke 2020; Pauls et al. 2022). In addition, other advances in MEG, for example, head movement compensation, pediatric chair, and customized arrays of OPM sensors, have made MEG studies of adolescents and even infants practical (Clarke et al. 2022; Feys and De Tiège 2024; Gaetz et al. 2014; Papadelis and Chen 2020).

When individual MRIs are unavailable, spherical conductor models are generally used as the simplest alternative (Berg and Scherg 1994; Mosher, Leahy, and Lewis 1999). Although they are more straightforward and faster to compute, they are known to provide less accurate source localizations than realistic head models, especially for sources in the frontal and temporal brain regions (Nummenmaa et al. 2013). Another alternative is to use a template MRI such as colin27 (Holmes et al. 1998) and ICBM152 (Mazziotta et al. 1995). However, using a fixed template MRI while the head shape and size varies across individuals reduces anatomical and geometrical fidelity, again providing compromised source reconstructions. This inaccuracy can be reduced by fitting the template MRI to the individual head shape determined, for example, by digitizing the scalp with an electromagnetic digitizer (Jaiswal et al. 2023). Three‐dimensional rigid‐body scaling of the template MRI to the head size is a simple and straightforward way to reduce the geometrical gap between the template and the individual head (Gramfort et al. 2013, 2014; Tadel et al. 2011); however, differences in the head shape remain and can cause substantial errors in the forward and inverse solutions.

Several methods have been proposed to resolve this problem by either selecting a matched MRI from a predefined database or by deforming a template MRI based on the individual head shape (Darvas et al. 2006; Gohel et al. 2017; Koikkalainen and Lötjönen 2004; Valdés‐Hernández et al. 2009; van't Ent, de Munck, and Kaas 2001; Yotter et al. 2011). van't Ent, de Munck, and Kaas (2001) utilized surface harmonic representations of different head surfaces to prepare a scalp, skull, and brain surfaces database. Given the digitized head shape and surface harmonics, the forward solution was computed with the best‐matching surfaces from the database. The authors demonstrated comparable model geometry and dipole fitting results using the proposed method and real individual MRIs. Koikkalainen and Lötjönen (2004) used free‐form deformation (FFD; Sederberg and Parry 1986) following an affine registration of a template surface set to generate a subject‐specific template set given the head digitization. Their study showed promising similarities between head surfaces reconstructed from the subject's MRI and estimated by deforming an average head surface; however, they did not present the effect on MEG/EEG source estimation. Valdés‐Hernández et al. (2009) proposed an approximate head model by estimating the average head shape from a target population of 305 subjects. The proposed method is computationally simple and estimates a better model than a generic template MRI. However, it relies on a specific population, does not consider individual head shape, and needs a large set of preprocessed MRIs from the target population. Later, Gohel et al. (2017) suggested a method for selecting a subject‐specific MRI from a pool of 92 segmented MRIs. Objective registration errors (OREs) were calculated for all scalp surfaces in the pool, and a single MRI or an average of 10 MRIs with the lowest ORE was chosen for the subject. They termed such a subject‐specific selected MRI as a pseudo‐MRI for the first time.

Darvas et al. (2006) used a thin plate spline (TPS; Bookstein 1989)‐based method and warped a template MRI to fit with the subject's head. They demonstrated that the warped template MRI reduced EEG source localization errors (LEs) by 14 mm on average compared to using a standard template and by 20 mm compared to the sphere model. The study utilized the locations of 155 uniformly distributed EEG electrodes as the head‐shape digitization to warp the Montreal brain template (Collins et al. 1998). Given such a set of dense and evenly sampled scalp digitization points, the TPS warping method computes a nonlinear warping transform for registering the template MRI's scalp to the individual scalp. Subsequently, this transform can be applied to other surfaces and anatomical derivatives of the template MRI. Although there is inter‐subject variation in brain structures at the subcentimeter level, the group‐level patterns, for example, distances between head surfaces, positions of functional regions, and underlying functional networks, are very similar within a specific age range (Douw et al. 2018; Fournier et al. 2011; Holliday et al. 2003). Thus, warping an appropriate template MRI with a correctly digitized scalp surface can provide the closest alternative to MRIs recorded from the individual's head.

Previous studies have offered substitute methods for establishing geometrical models when individual MRIs are unavailable. However, these methods have significant limitations in addition to the previously described study‐specific ones, and therefore, they have not been widely adopted by MEG and EEG users. The main shortcomings include:

1. *Requirement of a dense and even digitization*: These approaches are often limited to densely and evenly digitized scalps with generally more than 150 points. They still need to robustly address the practical limitations of the current digitization procedures, such as the existence of outlier points and sparsely or unevenly sampled scalp surfaces.
2. *Limited to scalp and skull surfaces*: Most approaches merely estimate surfaces required to define the conductor model; they usually do not warp the 3D MRI (voxels) or the cortical surfaces required for realistic visualization of source estimates.
3. *No simple user interface*: To the best of our knowledge, there is no easy‐to‐use software that seamlessly addresses the sparsity and unevenness of the digitized points and provides a 3D MRI stack along with the relevant surfaces for MEG/EEG source imaging.
4. *Non‐standard output format*: Some of the abovementioned methods are implemented within specific MEG/EEG analysis software packages. Since these packages employ a variety of file formats, coordinate systems, segmentation methods, and co‐registration procedures (Jaiswal et al. 2020), the output files cannot be readily transferred between the packages.

For example, the Brainstorm software (Tadel et al. 2011) implements the method by Darvas et al. (2006); however, the implementation requires evenly and densely sampled scalp digitization points, or it may fail to warp a template distortion‐free. Also, the output MRI is within a specific data structure, file format, and coordinate frame limited to Brainstorm; therefore, it cannot be readily used with other analysis software packages such as MNE‐Python (Gramfort et al. 2013, 2014), SPM (Litvak et al. 2011), or BESA (BESA Gmbh, Munich, Germany). The MNE‐Python package can also individualize a template MRI with a good user interface; however, the process is limited to 3D scaling. Additionally, none of these toolboxes handles uneven and sparse digitization to improve individualization. Although they remove points distant from the scalp, they do not check to ensure the distribution of points nor attempt to fill surface patches with no digitization points; therefore, their use is limited to the evenly sampled dense scalp digitization points. An even and dense point set would help TPS compute a smooth and tight‐fitting warping transform; however, the standardized digitization practice in MEG is user‐dependent (Jaiswal et al. 2023); therefore, a more robust method is required to efficiently work also with uneven and sparse digitization.

With the current study, we aimed to resolve the limitations of previous methods and implemented a tool that utilizes the TPS‐based warping method for generating the pseudo‐MRI based on scalp digitization that could also be uneven and sparse. Our tool, *pseudo‐MRI engine*, aims to improve over the previous methods in the following respects—it can work with inconsistent, uneven, and sparse scalp digitization; it provides all relevant head‐model surfaces for MEG/EEG source imaging as well as cortical parcellations; and it provides an easy‐to‐use interface and output files that can be used with many widely used analysis software packages.

To evaluate the performance of the tool, we examined the similarities between corresponding anatomical and source imaging metrics of 25 real and pseudo‐MRI sets. Similarities between head model surfaces, cortical surfaces, and canonical brain regions were examined. The source imaging metrics for each subject were also compared using simulated and real MEG responses employing three head models: the realistic‐shape models from the real and pseudo MRIs and a locally fitted sphere model.

## 2 Materials and Methods

### 2.1 TPS Warping

TPS (Bookstein 1989) is a commonly used interpolation technique that finds a “minimally bent” smooth surface passing through a given set of points. It is a conventional method for interpolating surfaces over a dispersed point cloud that is often sparse. TPS is also widely used for nonrigid image registration by matching the control‐point pairs. The TPS method is physically analogous to bending a thin metal sheet around the given point set (or surface). As the spline resists this bending due to its rigidity, it introduces some smoothness to the fitting surface (Bookstein 1989; Donato and Belongie 2002; Duchon 1977; Johnson and Christensen 2002; Luo et al. 2014). The bending introduces potential energy, referred to as *bending energy*, which is minimized while fitting the source surface to the destination points (or surface). The advantage of using TPS is that the deformations are local since the coefficients of the basis function defining the nonlinear warp can be effectively computed as a linear function of the control‐point positions (Carr et al. 1997; Darvas et al. 2006). The TPS warp in a three‐dimensional space with *N* control‐point pairs is described by $\left(N+4\right)\times 3$ parameters, comprising N×3 nonlinear warping coefficients and a 4×3 affine matrix for global affine motion parameters. These parameters are computed by solving a linear system.

In this study, TPS provides a mechanism to warp a template MRI to the subject's head. Subject's head digitization points, usually sampled with an EMT‐based digitizer (Jaiswal et al. 2023), and the scalp surface points of the template MRI are used to define the *control‐point pairs* to compute the warping transform. Keeping both point sets in the same coordinate frame, a selection of N digitization points, including LPA, Nasion, and RPA, are projected on the scalp surface of the template MRI to get the control‐point pairs; thus, each of the N pairs includes a digitization point and the corresponding point on the template scalp surface. Consider a three‐dimensional Euclidean space $\Omega \subset R^{3}$ in a subject's head coordinate frame with N pairs of control points $\left\{\left(p_{i},q_{i}\right)\;|\;i=1,2,3,\ldots ,N\right\}$, $p_{i}=\left(x_{i},y_{i},z_{i}\right)\in \Omega$, $q_{i}=\left(x_{i}^{,},y_{i}^{,},z_{i}^{,}\right)\in \Omega$, where $p_{i}$ is a control point on the template MRI's scalp corresponding to a digitization point $q_{i}$ on the subject's scalp. For an ideal warping, the point $p_{i}$ would move to match (overlap) with the corresponding $q_{i}$ points. These N pairs of control points define a warping transform f:Ω→Ω *for* mapping each position $\left(x,y,z\right)$ on the template MRI to a new position in Ω as.

(1) $$f\left(x,y,z\right)=a_{0}+a_{1}x+a_{2}y+a_{3}z+\sum_{i=1}^{N}w_{i}\left(\left|\left(x,y,z\right)-p_{i}\right|\right)$$

where $w_{i}\left(r\right)$ is the radial basis function of the warping transform, $w_{i}\left(r\right)\geq 0$. In Equation (1), $a_{0},a_{1},a_{2}$, and $a_{3}$ represent affine transformation coefficients, describing the global affine motion, and the latter part represents the nonlinear warping coefficients for the source control points *p*_*i*_. The affine transformation $a={\left(a_{0},a_{1},a_{2},a_{3}\right)}^{T}$, and the warping coefficients $\left\{w_{i}\;|\;i=1,2,\ldots ,N\right\}$ can be collectively written as a matrix $W={\left[w_{1},w_{2},\ldots ,w_{N},a_{0},a_{1},a_{2},a_{3}\right]}^{T}$ of dimension $\left(N+4\right)\times 3$ that must satisfy a linear equation LW=Q for matching the control‐point pairs.

The transformation coefficients are solved from Equation (1) by a linear solver method. Here, *L* is a nonsingular matrix of dimension $\left(N+4\right)\times \left(N+4\right)$, derived by the source control points on the template scalp as

(2) $$L=\left[\begin{array}{cc} K & P\\ P^{T} & Z \end{array}\right]$$

where Z is a zero matrix with dimension 4×4 and K is a positive definite matrix of dimension N×N computed as $k_{\mathrm{ij}}=w_{i}\left(\left|p_{i}-p_{j}\right|\right)$. Matrices P and Q are defined as

$$P={\left[\begin{array}{cccc} x_{1} & y_{1} & z_{1} & 1\\ \cdot & \cdot & \cdot & \cdot \\ \cdot & \cdot & \cdot & \cdot \\ x_{N} & y_{N} & z_{N} & 1 \end{array}\right]}_{N\times 4}Q={\left[\begin{array}{ccccccccc} x_{1}^{\prime } & \cdot & \cdot & \cdot & x_{N}^{\prime } & 0 & 0 & 0 & 0\\ y_{1}^{\prime } & \cdot & \cdot & \cdot & y_{N}^{\prime } & 0 & 0 & 0 & 0\\ z_{1}^{\prime } & \cdot & \cdot & \cdot & z_{N}^{\prime } & 0 & 0 & 0 & 0 \end{array}\right]}_{3\times \left(N+4\right)}^{T}.$$

Further, if there is a measurement noise, such as a few incorrect digitization points (outliers from scalp surfaces) or if the number of the destination control‐point pairs is too small to model the subject's scalp surface adequately, we need to relax the absolute warping by introducing a regularization parameter. The regularization parameter *λ* is a positive scalar that controls the level of smoothing in the warping (Donato and Belongie 2002) to avoid the overfitting problem. Thus, we compute the TPS coefficients by replacing the matrix K with K+λI in Equation (2), where I is the N×N identity matrix. Once the warping transform coefficients are computed by satisfying a linear equation LW=Q with the control point pairs, each voxel (or vertex) of the template MRI can be transformed to a new location using the warping transform W in Equation (1), as f:Ω→Ω. The bending energy is obtained by summing up the squared distances between the *N* pairs of control points. Increasing the number of control‐point reduces the bending energy in the TPS warp, indicating that a larger number of digitization points would provide a better warp.

**FIGURE 1:**
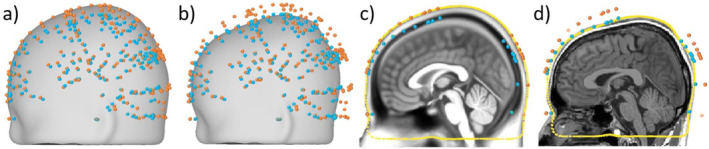
TPS warping of the MNI152 template MRI with 150 scalp digitization points (cyan color) from a 35‐year‐old adult subject: (a) A selection of control points (orange color) on the template scalp (gray color) corresponding to each digitization point; (b) warped scalp surface (gray color) after applying the TPS‐transform; (c) template scalp surface (yellow dashed line) and control‐point pairs overlaid on the template MRI; and (d) warped scalp surface (yellow dashed line) and control‐point pairs overlaid on the real MRI of the subject.

Figure 1a,b shows a typical case of warping a template MRI to a 35‐year‐old adult head. The MNI152 template scalp surface and the subject's scalp digitization points are shown in gray and cyan, respectively. The orange‐colored dots represent the closest points on the template scalp corresponding to each digitization point; they were determined by radially projecting the digitization points on the template scalp (see Figure 1a). The TPS transform was computed using the set of control‐point pairs and applied to generate the warped surface, as shown in Figure 1b. For a better 2D illustration, Figure 1c,d shows the sagittal cross‐sectional representation of Figure 1a,b around the midline.

### 2.2 Influencing Factors and Fine‐Tuning

The main factors that influence the warping of the template MRI are the digitization practice affecting the accuracy of the digitized points, the evenness and density of the point cloud, the selection of the template MRI, and the regularization parameter to optimize the warping.

#### 2.2.1 Accuracy of the Digitization Points

Digitization is an essential process usually performed during the subject preparation for defining coordinate transformations among head, device, and MRI; potential limitations may affect digitization accuracy (Jaiswal et al. 2023). Since the pseudo‐MRI engine utilizes the same digitization data as a pivotal input for warping the template, digitization density, distribution, and accuracy are crucial in generating pseudo‐MRI. The pseudo‐MRI engine employs heuristic approaches for ministering the digitization data and mitigating the chance of “loose or distorted warping” by employing the following steps: (i) selecting digitization points above a hypothetical plane approximately ~1 cm below the *fiducial plane* established by the nasion, left and right preauricular points (LPA and RPA); and applying the same selection for the template scalp vertices, (ii) selecting “good digitization points” by statistically identifying and omitting 2%–10% of the digitization points with extreme distances from template scalp, (iii) densifying the remaining digitization points if needed (see Section 2.3), (iv) limiting the digitization points under 300 by spatial downsampling to optimize the warping time, and (v) optionally, shifting the digitization points 1–2 mm inward to account for stylus elevation during scalp digitization caused by the stylus cap or dense hairs. The inward shifting helps to maintain a similar gap between digitization points and pseudo‐MRI scalp surface, as in the case of digitizing the subject's scalp in real. Although the tool applies these corrective steps, following the good‐practice guidelines for MEG/EEG digitization is advised, as Jaiswal et al. 2023) suggested previously.

#### 2.2.2 Effect of Digitization Density and Uniformity

**FIGURE 2:**
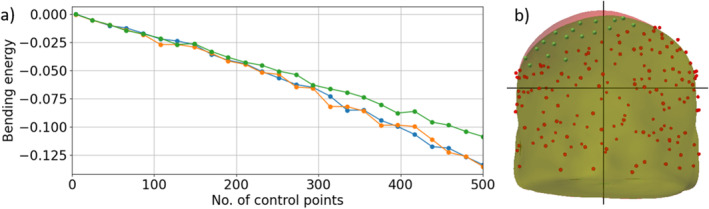
(a) The effect of scalp digitization density on bending energy. (b) Part of the head surface (red) was not correctly warped due to missing/omitted digitization points on a substantially large area.

The density and evenness of the digitization point over the subject scalp significantly impact the warping. A sparse digitized point set (< 50 points) allows only a lower number of control point pairs, requiring a higher regularization value and resulting in more relaxed warping with higher bending energy that may not accurately represent the subject's scalp. In contrast, extremely dense digitization points (> 500 points), such as those obtained by an optical scanner (Homölle and Oostenveld 2019; Zetter, Iivanainen, and Parkkonen 2019), prolong the warping procedure (see Table S1). Considering scalp surface area of an adult human is approx. 700 cm^2^ (Olsen and Canfield 2016), ideally 150–300 scalp digitization points (1–2 points per area of ~5 cm^2^) above the fiducial plane are suitable for fast and accurate warping. Figure 2a shows the change in bending energy with the number of uniformly distributed control points for three randomly selected subjects from the data set described in Section 2.5. The scalp data were reconstructed from the subjects' MRI. However, instead of using the real digitization recorded with the MEG data, we artificially generated a controllable, evenly sampled, dense digitization point set for this test. For that, we subsampled the scalp surface and inflated it by 2 mm, and added white noise (STD = 0.94 mm) to generate a realistic but uniform and dense point set. The point density in Figure 2a was varied by varying the subsampling. The plot (also Table S1) shows that the bending energy decreases with an increasing number of control points. Figure 2b depicts the effect of point uniformity, or in other words, the effect of an empty scalp region without a digitization point. The correctly warped green scalp surface was produced by warping the template scalp with evenly distributed 200 control points; bending energy = −0.040. However, the same warping produced a deviated surface (red) when the points around the left parietal region were omitted while computing the warping transform; bending energy = −0.340.

#### 2.2.3 Selection and Preparation of the Template MRI

Head shape, brain–scalp distance, and cortical structure vary across age, brain health conditions, and demography (Beauchamp et al. 2011; Fjell et al. 2015). Various MRI templates considering these factors have been made available by researchers, for example, colin27 (Collins et al. 1998; Holmes et al. 1998), MNI152 and ICBM (Fonov et al. 2009; Mazziotta et al. 1995), and NIHPD (Fonov et al. 2011). The pseudo‐MRI engine can ideally warp any template to a given set of scalp points; however, the template selection substantially impacts the congruence of the warped template with the subject's real MRI, especially for the cortical surfaces. Therefore, the closest representative template MRI should be employed to generate a pseudo‐MRI. For example, MNI152 is suitable for healthy adult populations rather than pediatric populations, while NIHPD is vice‐versa. For computing the warping coefficients, the template scalp surface and digitization points are kept in the same coordinate frame, and the closest point on the template scalp is selected for each digitization point. Therefore, the template scalp should be correctly reconstructed to match the anatomical features, avoiding any notable cavities on the surface. Figure S3 shows an example case.

#### 2.2.4 Effect of Regularization in Computing the Warping Transform

When computing the warping transform from the equation LW=Q, a regularization parameter λ is introduced to Equation (2) by replacing K with K+λI. It helps to overcome the problems with sparsely or nonuniformly digitized scalp points by controlling the level of smoothness in warping (Donato and Belongie 2002). Warping gets smoother with the increasing value of regularization, which becomes necessary when working with sparse control points. However, the surface produced by excessive regularization is substantially smoother and may differ significantly from the subject's scalp surface. On the other hand, the solver becomes unstable if the given regularization value is too small. Therefore, regularization should be data‐driven instead of a predefined value. As an improvement to earlier approaches, we implemented an adaptive regularization based on the given digitization data. We empirically tested and set an initial minimal value (i.e., 10^−10^), which adaptively converges to a higher value until the solver becomes stable. This heuristic always satisfies the linear solver, keeping the regularization minimal at the same time, and provides a tight (globally tight and locally smooth) warping even for control point pairs that are too sparse. This approach makes the pseudo‐MRI engine more robust for working with a broader range of digitization data.

### 2.3 The Pseudo‐MRI Engine Workflow

**FIGURE 3:**
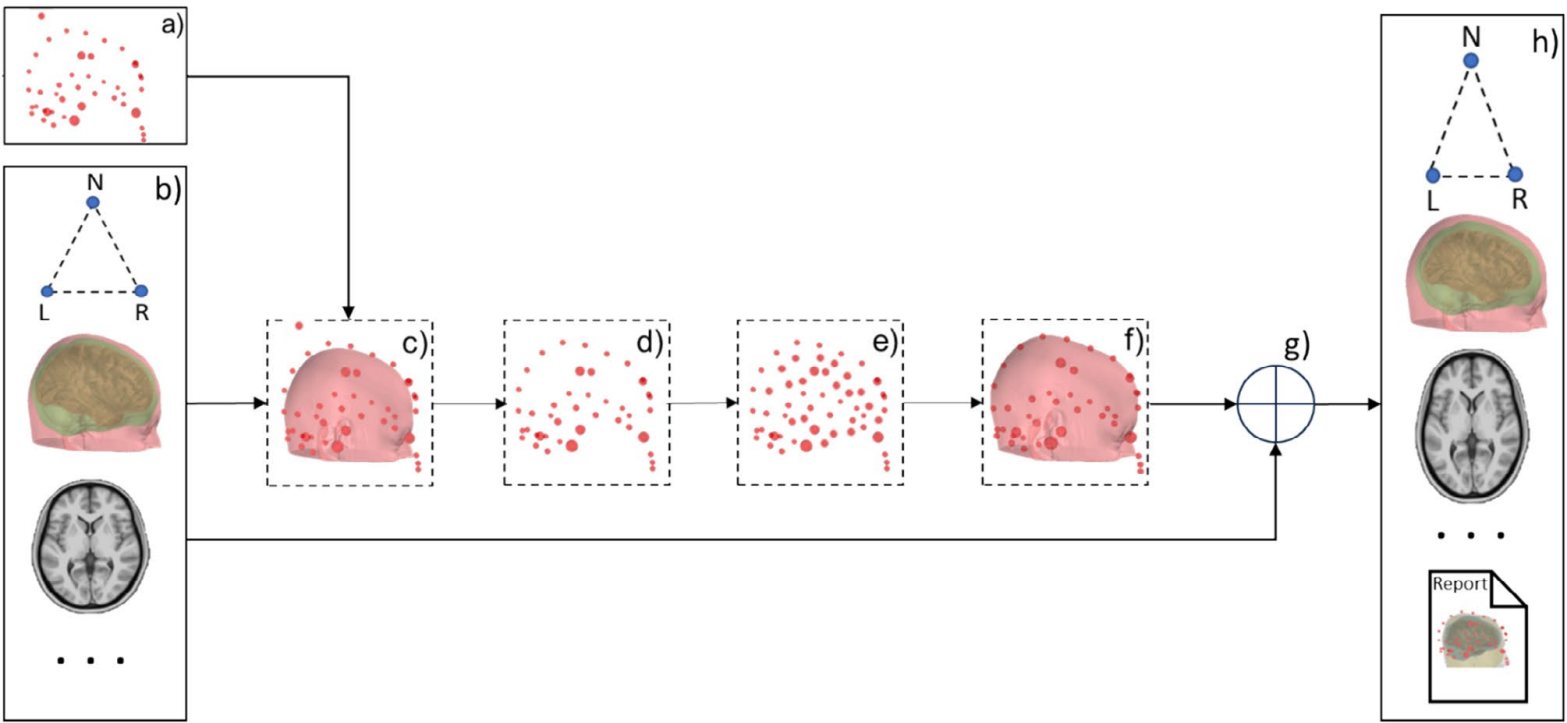
The pseudo‐MRI workflow: (a) Digitized headshape points (HSP), (b) segmented template MRI and matched fiducial points, (c) head‐MRI co‐registration, (d) HSPs outliers rejection, (e) heuristic densification of HSPs, (f) computation of warping‐transform with adaptive regularization, (g) application of warping transform, (h) template MRI files warped sequentially and saved to the pseudo‐MRI directory. N, L, and R represent nasion, LPA, and RPA fiducial points.

The *pseudo‐MRI engine* inputs a precisely segmented template MRI using FreeSurfer (or FastSurfer; Henschel et al. 2020; Henschel, Kügler, and Reuter 2022) and the subject's scalp digitization points, usually collected using an electromagnetic tracking (EMT) digitizer. The engine utilizes a three‐dimensional TPS warping method following a few novel heuristic approaches for rectifying and densifying the scalp digitization points. Figure 3 depicts the pseudo‐MRI generation workflow. Block *a* shows the digitized scalp or headshape points (HSPs) in the subject's head coordinate frame, including the three canonical fiducial landmarks. The segmented template MRI (MNI152; Mazziotta et al. 1995) is shown in Block *b*, along with the standard fiducial landmarks in the FreeSurfer (RAS) coordinate frame. Anatomically matched fiducial points for the template MRI are essential for accurate transformation and warping. Because the selection of LPA and RPA during digitization is user‐ or lab‐dependent (see Figure S4), using photographs or symbolic representations of fiducial point locations during digitization aids in choosing accurate fiducial points on the template MRI. We prepared three fiducial files for the template MRI for the three possible locations for LPA and RPA. The pseudo‐MRI engine, by default, selects the fiducial file with the commonly considered LPA/RPA locations, that is, *the helical crus‐tragus intersection point*. However, depending on the scalp digitization information, the user can choose a file that considers the *center of the tragus* or *the intertragal notch* for LPA and RPA. Please note, in the real use case, when there is no real MRI to get the scalp surface to match/fit with the scalp digitization points, we cannot correctly co‐register the template with just digitization points. Therefore, precisely matching the template MRI's fiducial points with the corresponding fiducials in digitization data is crucial for a good‐quality pseudo‐MRI.

At Block *c*, the HSPs are transformed to FreeSurfer's (surface‐RAS) coordinate frame and aligned with the scalp surface, using an MRI‐to‐head transform calculated by matching the fiducial landmarks from HSPs and template scalp. Block *d* filters HSPs, discarding those below a plane ~1 cm below fiducial points and detecting outliers based on scalp‐surface distance statistics, discarding at least 2% of HSPs. Block *e* checks and improves the uniformity and density of remaining “good‐quality” HSPs using either of two novel heuristic approaches: (i) *HSPs densification via TPS warping* and (ii) HSP *densification via sagittal mirroring*. The former selects the closest‐matching scalp surface from a predefined database, if available, based on HSPs‐scalp distance, warps and tessellates to a lower number of vertices, discards the points below ~1 cm to the fiducial plane, and merges new vertices to the original HSPs. The latter utilizes left–right head symmetry and mirrors the HSPs against the sagittal plane passing through the nasion, doubling point density without any predefined database. Block *f* computes the TPS warping transform to accurately warp the template scalp surface, using the HSPs and their corresponding nearest locations (radial projections) on the template scalp surface as *control points pairs*. Before computing the warping transform, the HSPs are shifted inward by a prefix value (~1–2 mm) to mimic the real digitization scenarios where the scalp surface typically stays below the HSPs rather than passing through them. The workflow uses the TPS approach akin to Darvas et al. (2006) and Luo et al. (2014); however, it incorporates adaptive regularization while computing the warping transform. It estimates N×3 warping coefficients, a 4×4 affine matrix (A), and computes bending energy (E) for *N* pairs of control points.

Further, at Block *g*, the warping is applied sequentially to all segmentation files of the template MRI (from Block *b*), and saved to the output directory following the same data structure, as shown in Block *h*. The MRI‐to‐head transformation (from Block *c*) and template scalp fiducial points are also warped and saved to the output directory, eliminating additional co‐registration requirements. Thus, the generated pseudo‐MRI comprises a complete set of FreeSurfer (or FastSurfer)‐reconstructed files individualized to the MEG/EEG subject. A configuration file containing the relevant parameters for computing the warping transform is written. In addition, an HTML report documenting steps from Blocks *a* to *h* is also written and saved so that we can review the warping quality at any time.

The study tested the warping of various MRI templates, including the MNI152 template MRI, and found that alternative templates, like the NIHPD template for a pediatric population or a specific template prepared for a particular group, can be similarly used. The selection of MRI templates can be based on factors like age range, scalp distance, and neurological factors. The pseudo‐MRI engine, which generates pseudo‐MRIs for individuals, does not require FreeSurfer/FastSurfer and can generate them quickly. Table S2 shows the time consumption by the engine in a typical operation of warping the template MRI on a Linux workstation.

### 2.4 Implementation and User Interface

The *pseudo‐MRI engine* is implemented in Python (version 3.8 and later) and is dependent on MNE‐Python (version 0.23 and later) and Nibabel (version 5.1.0 and later;; Brett et al. 2023). FreeSurfer (or FastSurfer) is used for segmenting the template MRI; however, it is no longer required for warping once the segmentation is done. The engine can be used via the command line or by a graphical user interface. The former is faster, less user‐dependent, and extensively tested and used in the study, while the latter provides an interactive way to use the tool. Figure S5a,b shows a typical command‐line script and the graphical user interface; Table S1 shows the CPU time usage with the command‐line interface on a test workstation.

### 2.5 Data Sets for Validation

To validate the performance of the pseudo‐MRI engine, T1‐weighted head MRIs, head shape digitization points, and MEG data from 25 healthy subjects were utilized. Of the 25 subjects, 23 were randomly selected from the CamCAN data set (Taylor et al. 2017) within the age range of 18–55 years. The other two subjects were also healthy adults within the same age range. These two subjects were taken outside of the CamCAN database to expand the validation test with data from two different systems located at two different sites to show the generalizability of the software, even though differences in acquisition system, site, and vendor should have minimal impact on the pseudo‐MRI generation. Visual, auditory, and somatosensory evoked responses were also recorded in these two subjects for validation beyond simulation. The informed consent was obtained from these subjects in agreement with the approval of the local ethics committee. The mean age across subjects was 29 years. The head‐model surfaces, cortical surfaces, and canonical functional regions were compared to examine the geometrical similarities between the real and pseudo‐MRIs. The differences at the source imaging level were examined using simulated and evoked MEG responses. To examine the discrepancy at the source level, source estimates using three different single‐compartment boundary element models (BEMs; Hämäläinen and Sarvas 1989) were compared: (i) realistically shaped BEM defined by the surface from the real MRI, (ii) realistically shaped BEM defined with the pseudo‐MRI surface, and (iii) an analytic BEM using a locally fitted sphere. The structural MRI, digitization data, simulated MEG, and human MEG evoked responses are detailed below.

#### 2.5.1 Structural MRI

The T1‐weighted structural MRIs for the 23 CamCAN data sets were recorded at MRC Cognition and Brain Sciences Unit, Cambridge University, UK, using a 3‐T MRI scanner. MRIs for the other two subjects were acquired at Helsinki University Central Hospital (HUS), Helsinki, Finland, using a 3‐T MRI system. These MRIs were segmented using the *recon‐all* routine in FreeSurfer software (version 7.2; Dale, Fischl, and Sereno 1999; Fischl 2012; Fischl, Sereno, and Dale 1999), creating MRI segments, cortical surfaces, and atlas‐mapped brain regions. Afterward, the scalp and the inner and outer skull surfaces were reconstructed from these MRIs using the watershed approach implemented in MNE‐Python. These surfaces were manually checked to be free of visible reconstruction errors.

#### 2.5.2 Scalp Digitization Points

The scalp poor HSPs were digitized during the subject preparation for MEG data acquisition using a Fastrak EMT system (Polhemus, Colchester, USA). 50–150 points defining the scalp surface of the subject's head were digitized following the three fiducial landmarks (LPA, Nasion, and RPA) and five head position indicator (HPI) coils. Figure S6 shows the density and uniformity distribution of the digitization points for all the subjects. Since we used retrospective cohort data sets, no special care was taken during digitization for the purpose of pseudo‐MRI generation. All the digitization points were transformed into the head coordinate frame defined using the fiducial landmarks and saved in the MEG data file as header information.

#### 2.5.3 Simulated MEG Evoked Responses

**FIGURE 4:**
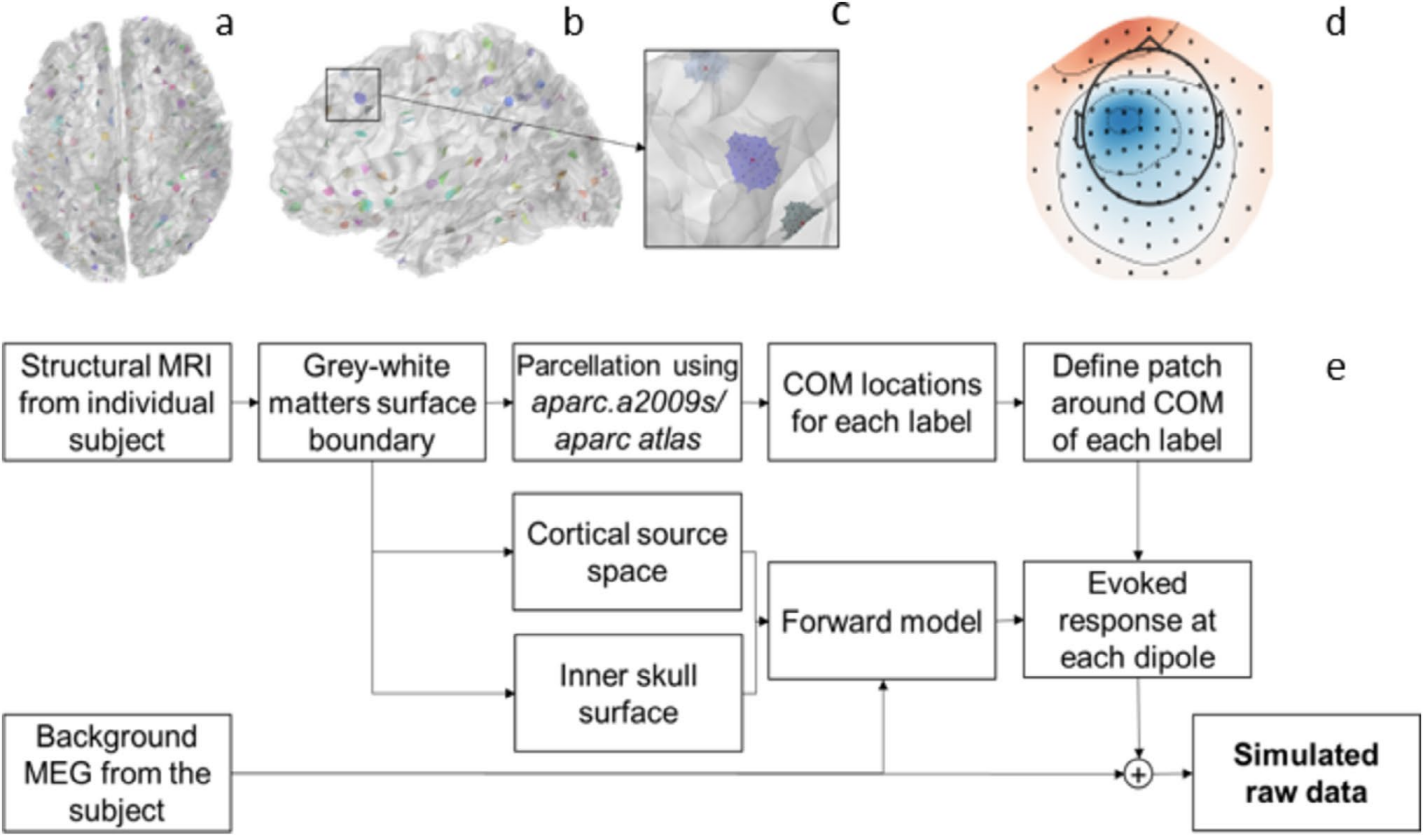
(a) Top view, (b) left view of dipole patches with aparc.a2009s parcellation and with patch size 25, (c) a magnified view of a dipolar patch, (d) sensor‐level simulated data field map for magnetometers for a source with *Q* = 400 nAm at peak ~75 ms, and (e) the simulation workflow.

We simulated sensor‐level evoked responses for all subjects using their real resting‐state MEG as background noise. The resting‐state MEG data for the 23 subjects (from the CamCAN database) were recorded with a 306‐channel Vectorview system (Megin Oy, Espoo, Finland) at MRC Cognition and Brain Sciences Unit, Cambridge University, UK, while the two other subjects were recorded using a 306‐channel TRIUX system (Megin Oy, Espoo, Finland) at Biomag laboratory of Helsinki University Central Hospital, Finland. We used sensor array information from resting‐state MEG recording, a single‐shell realistic volume conductor model based on the inner skull surface, and forward solutions for dipolar source patches to run the simulations for each subject. The conductor model was computed using the boundary element method (BEM; Hämäläinen and Sarvas 1989) with an “*ico4*” subdivision of the inner skull surface providing 2562 vertices and 5120 triangles with ~6 mm mean side length. The dipolar patches on the gray–white matter boundary of the real MRI were groups of a fixed number of adjacent vertices centered around the center of mass (COM) of 148 parcels following the Destrieux parcellation scheme (Destrieux et al. 2010). These patches were simulated—one at a time—with a 10‐Hz sinusoid of 200‐ms duration (two cycles) source activation, and the sensor‐level evoked responses were computed. The number of sources/vertices in the patches, hereafter referred to as patch size, was kept at 5, 10, or 25, and they were simulated at three amplitude levels: 50, 100, and 200 nAm. Figure 4a–d shows all dipole patches of size 25 as per *aparc.a2009s* parcellation for a subject. Figure 4d shows a sensor‐level simulated evoked response at the *left hemispheric frontal superior sulcus*, for the dipole with *Q* = 400 nAm and patch size 25. Figure 4e illustrates the complete simulation workflow.

#### 2.5.4 Human MEG Evoked Responses

Besides simulated MEG, we also utilized human MEG to examine the correspondence between the source imaging metrics when using BEM models from real and pseudo‐MRI. Evoked response MEG data from two healthy human subjects (35 and 52 years old) were recorded with a 306‐channel MEGIN Neuromag system (at BioMag Laboratory, Helsinki, Finland), while subjects received a random sequence of eight different categories of stimuli. These stimuli were visual (a checkerboard pattern in one of the four quadrants of the visual field), somatosensory (electric stimulation of the median nerve at the left/right wrist at the motor threshold), and auditory (1‐kHz 50‐ms tone pips to the left/right ear), with an inter‐stimulus interval (ISI) of ~500 ms. The stimuli were produced using Presentation software (Neurobehavioral Systems Inc., Albany, CA, USA).

#### 2.5.5 Pseudo‐MRI

Using the *pseudo‐MRI engine*, a pseudo‐MRI was also generated for each of the 25 subjects. The number of HSPs across the subjects ranged between 50 and 150 (see Figure S6). Since the retrospective cohort data sets were used, we could not manually identify the fiducial landmarks chosen during the digitization. The MNE‐Python co‐registration module was used to interactively match the HSPs to the subject's scalp surface from the real MRI and identify the fiducial locations. The *helical crus‐tragus intersection points* were found to be chosen as LPA and RPA during digitization in all the subjects. The fiducial files following similar locations for template MRI were used for co‐registration in the pseudo‐MRI generation (see Figure 3c). Considering the age range of the subject, the *MNI152* MRI template was used. The engine was run using the command line interface, with the segmented MRI template and HSPs. An HTML report file for each subject was generated and checked to verify and avoid any abnormal warping; we found no abnormal‐looking pseudo‐MRI across the subjects.

### 2.6 Comparison of the Pseudo‐MRI Engine to Other Methods

When warping a template MRI with sparse scalp digitization data using Brainstorm, we observed deteriorated outputs (see Figure S1). Furthermore, to test the performance of the pseudo‐MRI engine against such available similar tools, we compared it with Brainstorm's warping and MNE‐Python's scaling methods. An ICBM152 template MRI was adapted for five subjects employing Brainstorm‐warping, MNE‐Python scaling, and our pseudo‐MRI engine. These subjects were selected from the data set described in Section 2.5. Each of the subjects had ~150 digitized points, and the warping/scaling was performed for five levels of digitization densities—25, 50, 75, 100, and 150 points—by subsampling them from the existing digitization data. Points below the fiducials were removed before warping in Brainstorm, following 2% outlier rejection. In MNE‐Python, using the co‐registration GUI module, the template was scaled for all subjects using the five levels of digitization density, omitting points > 5 mm from the scalp. In the pseudo‐MRI engine, the warping was applied following several automated steps, such as removing points below a plane 5 mm lower to the fiducial points, checking the points distribution, and densifying them if the number remains < 50. Further, point (vertex)‐wise distances of pseudo‐MRIs inner‐skull surfaces from that of the real MRI were computed for all subjects at each digitization level. Consequently, the same was done for the Brainstorm‐warped MRIs and the scaled MRIs by MNE‐Python. These computations were also repeated for the scalp surface. Further, the warped/scaled MRI surfaces were compared with the corresponding real‐MRI surfaces, using the *t*‐test over the distance distributions.

### 2.7 Data Analysis

Anatomical and MEG source‐imaging comparisons were performed using head and cortical surfaces from the real and pseudo‐MRIs. For the MEG source imaging comparisons, single‐shell BEMs were utilized using the inner‐skull surface from the two MRI sets for each subject; a sphere model fitted to their scalp digitization points was also employed. We used *t*‐tests and other statistical measures to summarize our results from the comparisons utilizing the two MRI sets and the three BEM models. We did not apply a correction for multiple comparisons as the *p* values were used only as an aggregate (mean value) rather than declaring significance based on any of those *p* values falling below, for example, 0.05. For a simpler understanding, we used box‐violin plots to depict our findings. Plots were accompanied by tables with statistical metrics, including the mean value, interquartile range (IQR), outliers (𝑄3 × 1.5), and extreme (𝑄3 × 3.0) percentages. Better performance results from better stability, which is shown by a lower IQR value. For all the overlapping surface comparison plots, we have used green and red colors for the real and pseudo‐MRI surfaces, respectively.

#### 2.7.1 Computation of the Forward Models

**FIGURE 5:**
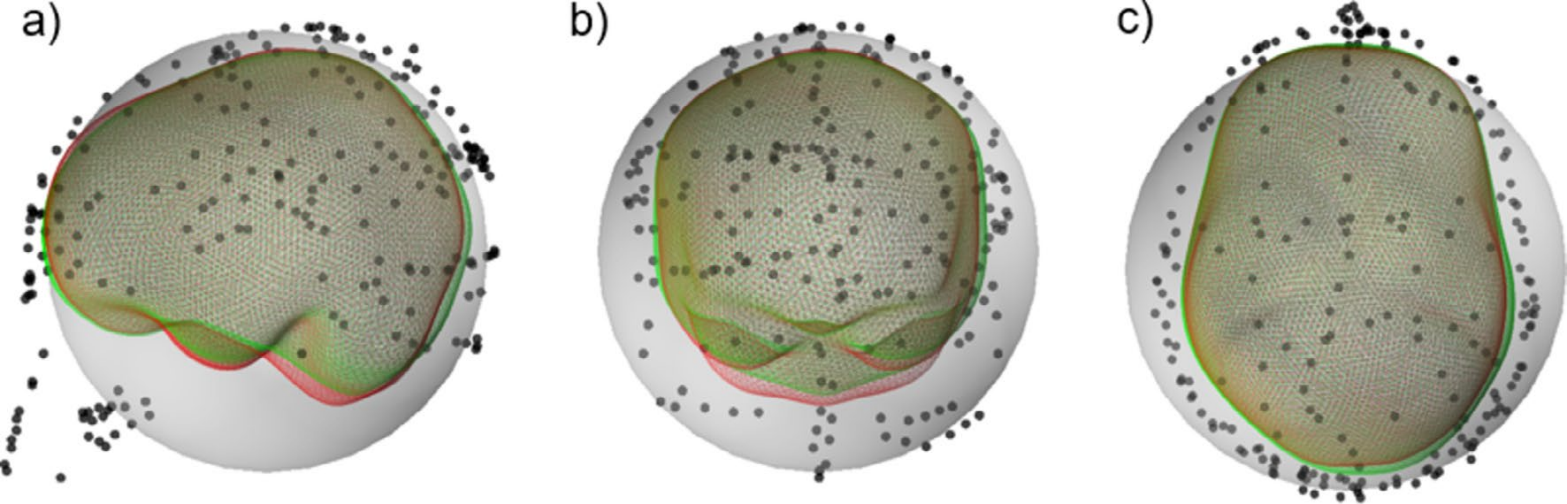
Single‐shell volume‐conductor‐model surfaces from the real‐MRI (green), pseudo‐MRI (red), and sphere (gray) fitted to the scalp digitization points (black): (a) left view, (b) front view, and (c) top view.

The scalp and skull surfaces are the essential information from the MRI for source imaging; they are utilized to define the realistic‐shaped BEM models for computing the forward solution. These surfaces are also used to constrain the source model. Therefore, to minimize the discrepancy in the forward solution, the BEM surfaces from the pseudo‐MRI should be closest to those from the real MRI. Figure 5 shows the three single‐shell BEM surfaces for an arbitrarily chosen subject, where the green and red surfaces are defined by the inner skull surfaces from the real and pseudo‐MRIs, respectively. The gray surface shows the single sphere fitted within the digitization points.

Further, we computed four different forward models using these conductor models, one for simulation and the other three for dipole fitting and beamforming scans. The forward model for the simulation was computed using the realistic‐shaped single‐shell BEM model (green) and cortically constrained source models, utilizing the real MRIs for subjects. The source models were obtained by *ico4* subsampling of the gray–white matter boundary and were oriented perpendicular to the local mantle. The other three forward models were computed for source estimation; they utilized an equispaced rectangular grid (volumetric source space) with 7‐mm spacing and enclosed within the three surfaces, as shown in Figure 5.

#### 2.7.2 MEG Preprocessing

MEG data were preprocessed with the Signal Space Separation (SSS) method, optionally including head movement compensation, using MaxFilter software (version 2.2; Megin Oy, Espoo, Finland). For the simulated MEG data, the SSS was performed on the background MEG file before using it in the simulation workflow (Figure 4e), whereas the human MEG response was performed on the raw MEG data file. Independent component analysis (FastICA; Hyvärinen 1999) was applied to the SSS‐preprocessed continuous data to mark eye‐blink and cardiac components above the 60% correlation threshold. These artifact components were rejected after visually inspecting their morphologies and the field maps to reconstruct the artifact‐free continuous data. After that, the SSS‐preprocessed continuous data were filtered using a zero‐phase filter with a 2–45 Hz passband and segmented into trials for −200 to +200 ms relative to stimulus onset. Using the variance‐based automatic trial rejection (Jaiswal et al. 2020), the trials with cross‐channel maximum variance higher than the 98th percentile of the maximum or lower than the 2nd percentile were rejected. The noise and data covariance matrices were computed using pre‐stimuli (−200 to 0 ms) and post‐stimuli (0 to +200 ms) segments, respectively.

#### 2.7.3 Comparison of Anatomical and Source Imaging Metrics

**FIGURE 6:**
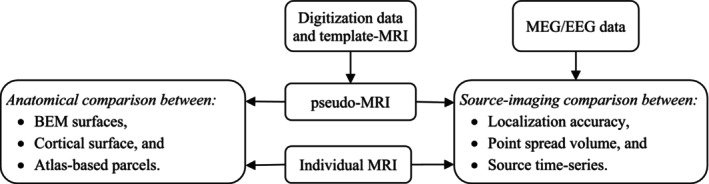
Analysis workflow for validating the congruence between the individual (real) and pseudo‐MRIs.

Figure 6 outlines the analysis to examine the congruence between the two sets of MRI. The anatomical comparison was performed by comparing the volume conductor model surfaces, cortical surfaces, and the atlas‐based parcels from the two sets for each subject. Using the following Equation (3), we computed the mean point‐to‐point distance $D_{r-p}$ for the two surfaces in the head‐coordinate frame, where $r_{r}^{i}$ and $r_{p}^{i}$ are *i*th point positions on the real and pseudo‐MRI surfaces, and $\left|r_{r}^{i}-r_{p}^{i}\right|$ is the Euclidean distance between them. N is the total number of vertices on both surfaces.

(3) $$D_{r-p}=\frac{1}{N}\sum_{i=1}^{N}r_{r}^{i}-r_{p}^{i}$$

For examining the source‐imaging metrics, we compared the sources reconstructed by ECD or LCMV approaches with the three conductor models, as shown in Figure 5. The sources were estimated for the simulated evoked responses at COMs of each 148 parcels. The evoked response sources for visual, auditory, and somatosensory stimuli were constructed from the human MEG response. We computed the LE in each estimate to examine the effect of using a pseudo‐realistic BEM model instead of a realistic BEM or sphere model. In addition, the point spread volume (PSV) and correlation between source (virtual) signals were also computed and compared for the three corresponding estimates. Please note that the scanning with realistic BEM used the same surface geometry as the simulation but a differently spaced (*ico4*) triangular mesh. Figure S7 illustrates the side lengths of surface meshes with *ico4* and *ico5* tessellation.

- *Comparison of BEM surfaces*: In MEG source imaging, only the inner skull surface is often used to compute the BEM, while EEG analysis uses the inner skull, outer skull, and scalp. The FreeSurfer watershed algorithm constructs triangular surface meshes for the brain, inner skull, outer skull, and outer skin (scalp), consisting of 10,242 vertices and 20,480 triangles. These surfaces are often tessellated with *ico4* or *ico5* spacing while defining the BEM. Since these vertices are identically arranged for all subjects, we computed the distance $D_{r-p}$ between corresponding vertices from the two surface sets for all four surface pairs using Equation (3). The distance should be minimal for a higher congruence between corresponding surfaces.
- *Comparison of cortical surfaces*: We found a high mean distance for the cortical surfaces when computing in Equation (3). This is because of the intrinsic difference between the corresponding surfaces from the real and pseudo‐MRIs. Since the template MRI is constructed by averaging MRIs from many individuals, usually within an age range, the cortical structures with high variability are averaged out. Thus, a template MRI may have fewer cortical foldings than an individual one. Although a pseudo‐MRI globally appears similar to the real one, its (local) cortical morphology may be substantially different. Therefore, instead of comparing the cortical surfaces, we compared the envelopes (concave hull), tightly enclosing the cortical surfaces. We also computed the Hausdorff distances (Taha and Hanbury 2015) between the corresponding surfaces from the two MRI sets by placing them in the head coordinate frame of the subject.
- *Comparison of atlas‐based parcellated regions*: We compared parcellated cortical surfaces from the two MRI sets to test the congruence between the brain regions. The parcellation scheme using the Destrieux atlas (aparc.a2009s; Destrieux et al. 2010) divides each hemisphere into 74 parcels, resulting in 148 parcels in total. Since these parcels are mapped over cortical surfaces, a parcel from a pseudo‐MRI usually has fewer vertices than the same from a real MRI; therefore, the point‐to‐point distance is not appropriate for the comparison. However, since the COM of a parcel is usually considered to represent its activity while data reduction, we compared their position and depth from the inner skull surface. We also examined the surface area and volume similarities of each parcel between the two MRI sets.
- *Comparison of LEs*: The true source COM locations were known for the simulated MEG data and were considered as reference locations for computing the LEs. For human MEG responses where the true locations were unknown, we used the *Source Modeling Software* (XFit; Megin Oy, Espoo, Finland) to fit a single dipole (ECD; Sarvas 1987) for each evoked‐response category at the time point around the peak of the average response, providing the maximum goodness‐of‐fit value. These locations were used as the reference source locations for the comparison. The ECD and beamformer estimates (LCMV;; Van Veen et al. 1997) were employed using MNE‐Python to determine the source location. Then, the LE was computed as the Euclidean distance between the reference location $r_{\text{true}}$ and the estimated source location $r_{\mathrm{est}}$, as $\mathrm{LE}=\left|r_{\text{true}}-r_{\mathrm{est}}\right|$.
- *Comparison of point‐spread volume (PSV)*: An ideal spatial filter should yield a unit response at the true source position and no activity elsewhere. However, spatial filter leakage spreads the estimates to surrounding locations because of noise, limited spatial selectivity, and regularization. The estimated source's focality (or focal width) depends mainly on the source's strength, direction, and proximity to the sensors. PSV, as a measure of the focality, is defined as the entire volume occupied by source activity above a threshold (Jaiswal et al. 2020). A lower PSV value thus means a more focused source estimate. We set a threshold of 50% of the highest activity in a source estimate to compute the PSV in the study. The volume that a single source in this study represents (5‐mm grid spacing) was 125 mm^3^. The PSV was computed by summing up the volume of all voxels above the activation threshold.
- *Comparison of source time series from pseudo‐MRI and individual MRI*: The correlations between estimated and simulated source time series were also compared to examine the congruence between sources reconstructed using the three different BEM models. A high correlation shows a smaller effect of the alternate conductor model. A higher correlation between the source time series using pseudo‐MRI and the one using real MRI indicates the possible use of pseudo‐MRI for functional connectivity estimates. However, functional connectivity‐based comparisons were not conducted in this study. *T*‐tests were applied to investigate the statistical significance of the congruence between the time series.

## 3 Results

### 3.1 Performance of Pseudo‐MRI Engine Compared to Similar Methods

The distance distributions, overlapping surfaces, and *T*‐statistics comparing the performance of the three methods at different digitization densities are shown in Figure S2, where plots (a–c) illustrate the findings for the inner skull and (d–f) for the scalp surface. The distance distributions for both surfaces indicate that the Brainstorm‐warped MRIs were notably different from the real MRIs until the warping was performed with 100 or more scalp digitization points. At lower point densities, the overlapping surface plots also show some distorted parts for the Brainstorm warping. The MNE‐Python scaling created more congruent MRI surfaces than the Brainstorm‐warping when fewer than 100 scalp points were used for warping/scaling. The pseudo‐MRI engine outperformed the former methods and generated highly congruent MRI surfaces at each density level. The distance distribution showed the lowest mean and IQR values for the pseudo‐MRIs in all the tests, indicating their high congruence with the real MRIs and also the robustness of the pseudo‐MRI engine. Furthermore, the *t*‐test among the surface's distance from the real MRI surface showed that the difference between pseudo‐MRI and the real MRI surfaces was significantly lower than that for the Brainstorm‐warped surfaces (*t* < −50, *p* < 0.01) and MNE‐Python‐scaled surfaces (*t* < −20, *p* < 0.01).

### 3.2 Anatomical and Source‐Imaging Similarities Between the Real and Pseudo‐MRIs

#### 3.2.1 Comparison of Real vs. Pseudo‐MRI BEM Surfaces

**FIGURE 7:**
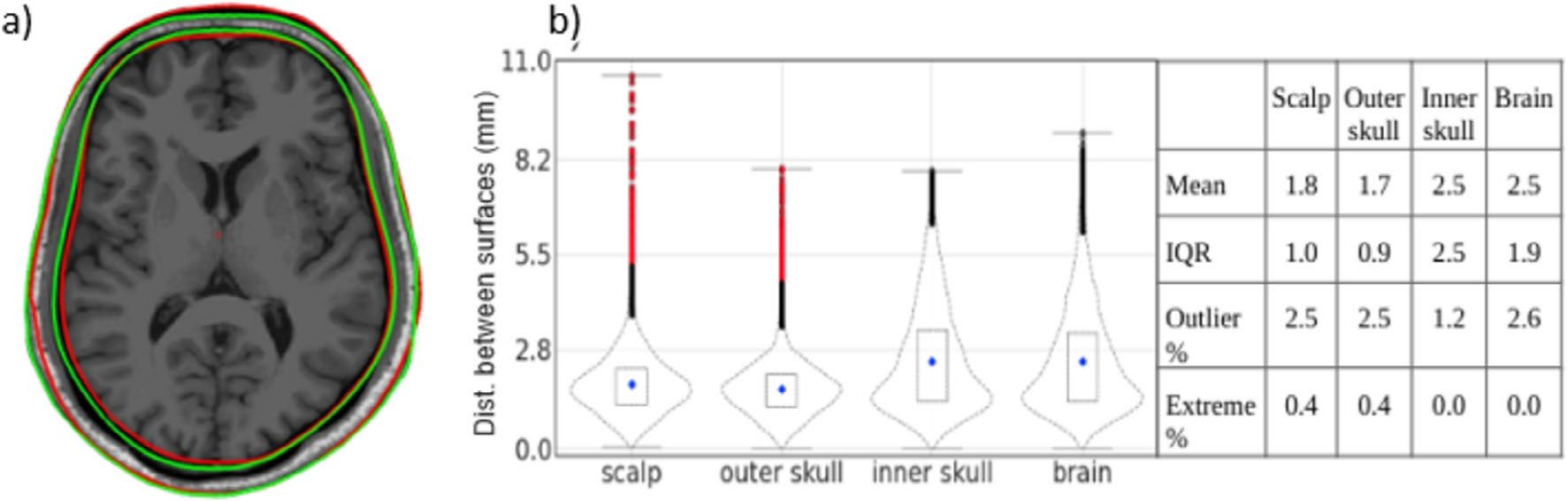
Conductor‐model surfaces for the real and pseudo‐MRIs: (a) The correspondence between the scalp and inner‐skull surfaces from the real (green) and pseudo (red) MRIs; the surfaces are overlaid on the real MRI. (b) Distribution of the point‐wise distances between the real and pseudo‐MRIs' surfaces and the distribution statistics.

Figure 7a shows the congruence between corresponding model surfaces, brain, inner skull, outer skull, and scalp from the real (green) and pseudo (red) MRIs for an arbitrarily chosen subject. The violin plots in Figure 7b show the point‐wise distances between 10,242 vertices of each surface pair, and the attached table lists the statistical measures of the distribution. The mean distance between corresponding surfaces for all the surface types was under 3 mm, showing high geometrical similarity and alignment. The outlier percentage, extreme percentage, and IQR for all four cases are also relatively low, which indicates higher stability in the findings. Some vertex pairs show higher distances but mainly appear on the inferior side. Some higher distances were also observed in the prefrontal region, possibly due to Yakovlevian torque (Fournier et al. 2011), which is the tendency of the human brain to exhibit a global, counterclockwise torque about the axis parallel to the long axis of the human body.

#### 3.2.2 Comparison of Cortical Surfaces

**FIGURE 8:**
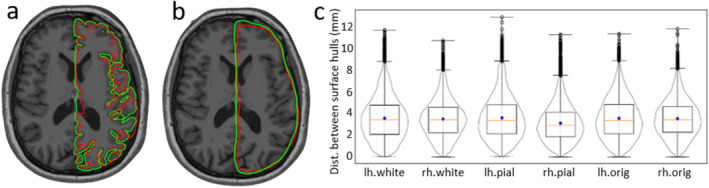
Comparison of cortical surfaces: (a) Pial surface, (b) pial surface envelops (hulls) from real (green) and pseudo (red) MRI of a subject, and (c) distribution of distances between cortical surface envelops from the real and pseudo‐MRIs.

When comparing the cortical surfaces from the real and pseudo‐MRIs (see Figure 8a), the surfaces appeared quite aligned and congruent; however, the mean distances between the surfaces were very high (~30–40 mm). To analyze their global congruence, we computed a concave hull for each surface in both hemispheres and then calculated $D_{\left|r-p\right|}$ for corresponding envelopes. Figure 8b shows overlapping pial surface plots for the two sets of MRIs of a subject, and Figure 8c shows the distance distribution *pial*, *white*, and *orig* surface hulls for left (*lh*) and right (*rh*) hemispheres. We found the mean distance $D_{\left|r-p\right|}<4\;\mathrm{mm}$, IQR < 3 mm, outlier < 2%, and extreme < 1%. Overall, the two surfaces are aligned and of equal shape and size, but some distant vertices were observed in the prefrontal area and inferior region. The Hausdorff distance distribution for the cortical surfaces is shown in Figure S8.

#### 3.2.3 Comparison of Atlas‐Based Segmented Regions (Parcels)

**FIGURE 9:**
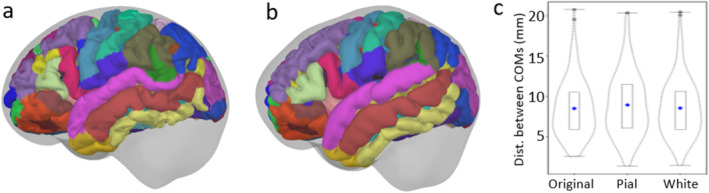
Comparison of the parcellated brain based on Destrieux atlas: (a) Real MRI parcels and (b) pseudo‐MRI parcels overlaid with their inner skull (gray) surfaces for a subject; and (c) distance between COMs for the two sets of parcels.

Figure 9 depicts the similarity between the segmented parcels of the real and pseudo‐MRIs, based on the Destrieux atlas (aparc.a2009s). Figure 9a,b shows the atlas regions for pial surfaces, while Figure 9c plots the distribution of the distance between the COMs of the 148 parcels for three surface types. The boundaries appear similar in the corresponding pairs; however, since the fine structure of the template cortex was averaged out, the template's parcels appear smoother. The mean distance between 148 COM pairs for all three surface types was approximately 8.5 mm, with the IQR ≈ 5.1 mm. When testing the COMs depth from the inner skull surface for the pseudo‐MRIs, we found high similarity (*ρ* = 0.90, *t* = 0.35, *p* = 0.88) with that of the real MRI (see Figure S9). Figure S10 shows the high similarities in surface area (*ρ* > 0.95) and volume (*ρ* > 0.75) between the parcels from the two MRI sets.

#### 3.2.4 Comparison of Single‐Dipole Fits/Models

**FIGURE 10:**
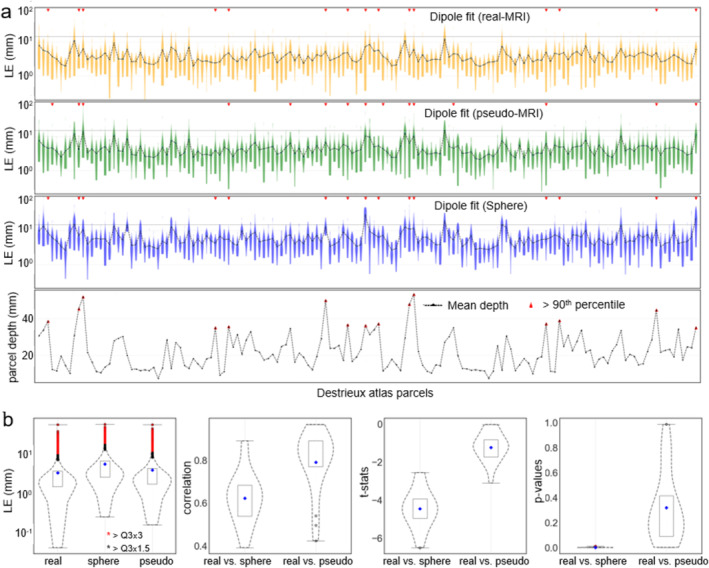
(a) Dipole localization errors for the simulated sources with a patch size of 10 dipoles at 100 nAm; (b) mean localization errors with the three models, and the correlation, *t*‐statistics, and *p* values for the two sets of comparison over the LE distributions.

We estimated the location of the simulated dipoles by using dipole fitting in MNE‐Python at the time point of the maximum RMS value across the planar gradiometer channels (global field power) of the average response amplitude. Figure 10a shows the LE distribution for all the simulated data across the 25 subjects for three BEM models, and the lowermost plot shows the mean COM depth across the subjects. Figure 10b summarizes the statistical characteristics of the LE distribution, showing lower LEs for realistic and pseudo‐realistic BEM models than those with the sphere model. A higher similarity (*ρ* = 0.81, *t* = −0.98, *p* = 0.38) was observed when comparing the LEs using pseudo‐realistic and realistic BEM. In contrast, a significant difference (*ρ* = 0.62, *t* = −4.4, *p* = 0.03) was observed between LEs using realistic and sphere models. Figure S11 shows the LE distribution of dipole fitting with evoked MEG response simulated for different dipolar strengths and patch sizes.

#### 3.2.5 Comparison of LCMV Location Estimates

**FIGURE 11:**
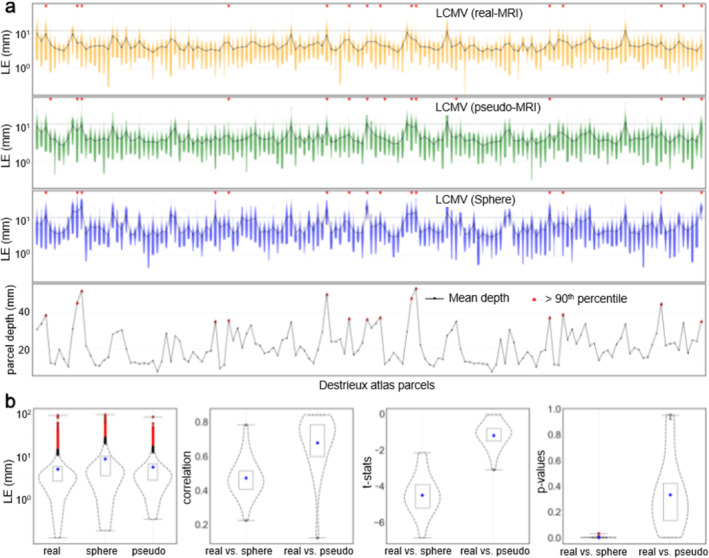
(a) LCMV source localization error for the simulated dipole patches of 10 sources at the amplitude 100 nAm; (b) correlation, *t*‐statistics, and *p* value computed by *t*‐tests for the same simulated data sets.

Figure 11 shows the distribution of LEs estimated by LCMV using three conductor models. When source‐to‐sensor distance increases, the signal attenuates; therefore, the probability of mislocalization is higher in the case of deeper sources. The statistical metrics for source estimates with pseudo‐realistic BEM (*ρ* = 0.64, *t* = −0.91, *p* = 0.37) are similar to those estimated using a realistic BEM. In contrast, the source estimate using a sphere model showed a lower similarity (*ρ* = 0.46, *t* = −4.88, *p* = 0.05).

#### 3.2.6 Comparison of LCMV Focality Estimates

**FIGURE 12:**
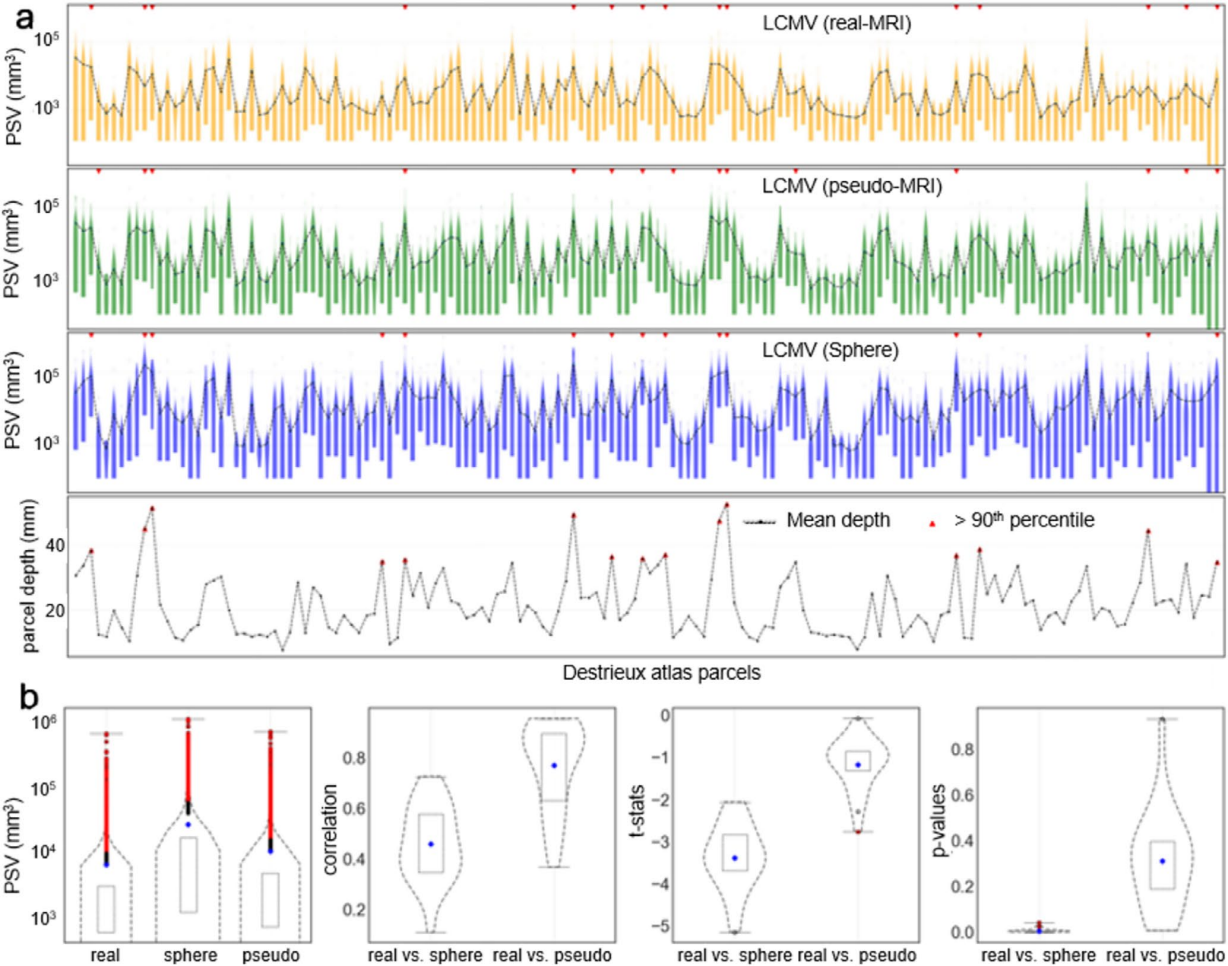
Point spread volume for the LCMV source estimates: (a) PSV distribution for the source estimates with simulated data across the subjects; (b) the mean PSV, correlation, *t*‐statistics, and *p* values for the comparisons.

For each LCMV source estimate, the PSV was computed in *cubic millimeters* by multiplying the number of active voxels with the volumetric representation of one voxel ($125\;mm^{3}$). Figure 12a demonstrates the PSV distribution for the three BEM models against the parcel (COM) depths. Figure 12b shows the mean and other statistical measures explaining the similarity between realistic and pseudo‐realistic BEM models. It shows similarities (*ρ* = 0.79, *t* = −1.21, *p* = 0.36) between PSVs using pseudo‐realistic and realistic BEMs. In contrast, a significant difference (*ρ* = 0.46, *t* = −3.49, *p* = 0.01) was found between the PSV estimated using the sphere and realistic BEM.

#### 3.2.7 Comparison LCMV Source‐Time Series

**FIGURE 13:**
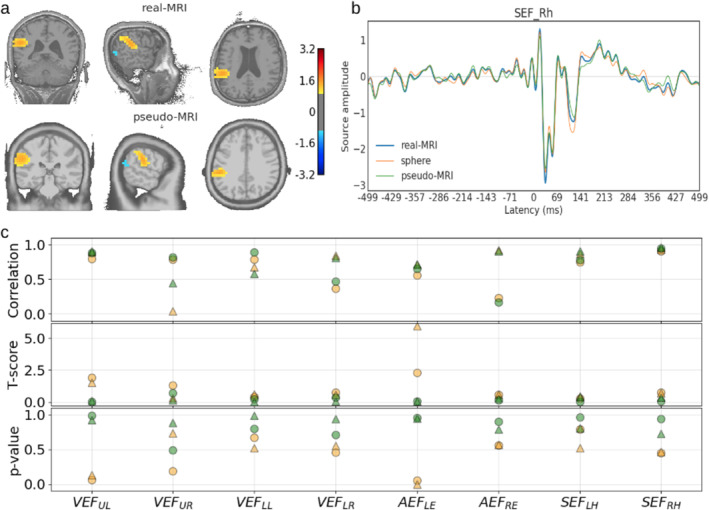
(a) Right wrist stimulated SEF MEG response for a subject estimated with real and pseudo‐MRIs; (b) similarity among the peak source activities estimated with the three conductor models; (c) correlation and *t*‐test metrics for visual, auditory, and somatosensory evoked responses' peak activities from two subjects; the orange and green colors, respectively, show the values for comparison of sphere and pseudo‐MRI based estimates with the one with real MRI. The two markers represent values from the two individual subjects.

After estimating the source locations using LCMV spatial filters with the three conductor models, we computed the source time series (virtual signals) at the true locations. We then calculated the correlation between the sensor‐level signals and the estimated source time series computed using the three BEM models. Overall, the highest correlation (ρ>0.95) was observed for the source time series estimated using the realistic BEM. The pseudo‐realistic BEM models also showed a similar level of correlation (ρ>0.93). In contrast, the estimates with sphere models showed comparatively lower correlation values. Figure 13 shows the similarities between source time series from the same source but with the LCMV filter with different conductor models. Here, VEF, AEF, and SEF stand for visual, auditory, and somatosensory evoked fields, respectively; UL, UR, LL, and LR show whether the visual stimuli were presented at the upper left, upper right, lower left, or lower right quadrant of the visual field. LE and RE indicate whether the audio stimuli were delivered to the left or right ear, and finally, LH and RH stand for left and right hands for the electrical nerve stimuli.

## 4 Discussion

The comparison of the pseudo‐MRI engine with Brainstorm's warping and MNE‐Python scaling shows that our software outperforms the available methods, especially for sparse scalp digitization. The results indicate that Brainstorm may provide unreliably warped MRIs when there are less than 100 scalp digitization points; however, given a dense digitization of more than 150 scalp points, both Brainstorm‐warping and MNE‐scaling provide reliable output. The automated steps applied to the digitization data in the pseudo‐MRI engine before computing the warping transform and the adaptive regularization ensure and improve the generation of pseudo‐MRIs for a wider range of digitization density. Although the MNE‐Python‐scaled MRI sometimes appears similar to the pseudo‐MRI, its distance distribution's IQR is higher than that of the pseudo‐MRI, indicating comparatively loose fitting with the subject's head. Therefore, among the tested methods, the pseudo‐MRI engine generates the closest alternative to the individual's real MRI with the fewest scalp digitization points.

Further, the study compared the anatomical and source‐imaging metrics when using pseudo‐MRI with real MRI. The mean differences between corresponding BEM surfaces were found to be < 2.5 mm. Although it is low, there are also other factors in MEG/EEG source imaging that may affect spatial accuracy, such as co‐registration, head movement, and MEG/EEG preprocessing. Differences in cortical surface geometries were found to be substantially high; however, the *between‐subject* variability in brain structure is a known fact (Fjell et al. 2015). Therefore, the cortical source mapping with pseudo‐MRI may sufficiently deviate from the one with real MRI. We recommend using a pseudo‐MRI for a volumetric source space rather than a cortically constrained one. Nevertheless, the differences between the corresponding surface envelopes (concave hulls) from the real and pseudo‐MRIs were relatively small. It indicates that if we ignore the microscopic morphometric difference between the cortical surfaces, the pseudo‐MRI surfaces are closely aligned with the corresponding surfaces from real MRI, indicating a subcentimeter spatial similarity between them.

The comparison between atlas‐based cortical parcellation also showed subcentimeter spatial similarity. Since the gyral pattern of a cortical surface is averaged out in an MRI template based on averaging multiple subjects, a side‐by‐side comparison between corresponding parcels from the real and pseudo‐MRI was not reasonable. However, since COM represents the kernel of a parcel and is often chosen to extract the source activity from the parcel, a comparison can be made between the distances of parcels in real and pseudo‐MRI. Also, the depth of corresponding parcels from the two sets was compared. The mean distance between corresponding COMs was < 1 cm with an IQR of 5.1 mm, and the parcels' depth from the inner skull surface were very similar. It indicates the possibility of using pseudo‐MRI parcels as an alternative to the ones with the real MRI, with a spatial uncertainty of ~1 cm.

Previous studies have shown higher inaccuracy in LCMV source estimates for deeper sources due to low SNR (Jaiswal et al. 2020; Nenonen et al. 2022); a similar pattern can be seen here, too. However, when comparing against the single dipole localization using a realistic BEM model, the localization using pseudo‐realistic BEM showed greater similarity (*ρ* = 0.81, *t* = −0.98, *p* = 0.38) than the ones using the single sphere model (*ρ* = 0.62, *t* = −4.4, *p* = 0.03). It indicates the pseudo‐realistic BEM as a better alternative than a single sphere fitted with a head shape. Similar patterns were also observed when comparing the LCMV estimates, with greater similarities (*p* = 0.37) between pseudo‐MRI and real MRI than between sphere and real BEM (*p* = 0.05). As expected, when comparing the focality pattern of the sources estimated using realistic and pseudo‐realistic BEMs, we found similarities (*p* = 0.36), in contrast to a significant difference (*p* = 0.01) between focality patterns using sphere and realistic BEM. Furthermore, the source locations and time courses for human MEG evoked‐responses estimated using pseudo‐MRIs were consistent with the ones obtained with the real MRIs. Some of the VEF and AEF responses appeared less consistent than SEFs, which could be due to their lower consistency in general. Such inconsistency was also observed in our previous study (Jaiswal et al. 2020).

We observed high anatomical similarities between the real and pseudo‐MRIs. The MEG source imaging results suggest that the pseudo‐MRI could substitute for the individual MRI when subcentimeter spatial accuracy is not required. This paper presents the methodology, software implementation, and validation over the adult age group. As pediatric brain morphology is significantly different from that of the adult brain, it would be required to validate the software for pediatric cases. Also, the current study presents results using digitization points collected by the Fastrak EMT‐based digitizer. However, digitization from other EMT‐based alternatives like NDI Aurora (Jaiswal et al. 2023) or scalp point‐cloud scanned using a 3D scanner, for example, Structure IO, can also be used similarly for generating pseudo‐MRI. Further, optical scanners like Structure IO are getting more common for EEG and MEG, especially OPM MEG, so it would be valuable to present a validation over optical digitization‐based pseudo‐MRI generation in the future.

Although the heuristic approaches used in the study improve the warping and thus the pseudo‐MRI, careful and correct scalp digitization is the key to improving the pseudo‐MRI following the guidelines from (Jaiswal et al. 2023). The pseudo‐MRI engine requires segmentation and surface correction of an MRI template only once, which saves time and effort in running the analysis for subjects in the studies. The study was performed with retrospective data where the digitization process potentially deviated from the guidelines suggested by (Jaiswal et al. 2023). Thus, the results show the general performance of the tool, which can be further improved if digitization is done more carefully to define the subject's scalp with uniformly distributed dense point sets.

The geometrical and source‐imaging similarities between the real and pseudo‐MRIs advise that the pseudo‐MRI generated by our engine is very close to the real MRI and can be used in MEG source imaging. It can replace the need for the time and resource‐consuming MRI scan and its preprocessing. However, it can only be used for applications where subcentimeter spatial accuracy is unnecessary. Such a pseudo‐MRI can also be used for quality control during data acquisition by verifying the locations of the elicited response at the source level. It can also help monitor the movement inside the MEG using a patient‐specific virtual head. Further, a pseudo‐MRI generated with an appropriate pediatric template can support electromagnetic source imaging in pediatric populations where MRI scans are often challenging due to lower noise intolerance, higher head movement, and greater ethical restrictions when scanning very young children. In addition, the pseudo‐MRI supports data privacy and allows users to share the surrogate anatomical data with fewer restrictions (Vinding and Oostenveld 2022).

Although the pseudo‐MRI engine provides a viable alternative to the individual MRI, it is not recommended for MEG applications requiring subcentimeter spatial accuracy. The current version of the pseudo‐MRI engine is validated only with the data from a conventional digitizer; however, the software can ideally generate pseudo‐MRIs from 3D scalp points recorded with any hardware, given that the points are in the head coordinate frame and fiducial landmarks are correctly defined. Considering the growing potential of more economical alternatives, especially in EEG (Homölle and Oostenveld 2019), the software should be validated with the 3D‐scanned vertices from optical scanners such as Structure IO (Occipital Inc., USA). Although the study investigates source imaging using the pseudo‐MRI approach only for MEG data, the approach is likely very useful also for EEG source imaging, where it could potentially provide a localization accuracy similar to that obtained with forward models based on real MRIs. Since the pseudo‐MRI engine utilizes a precisely segmented template MRI, the pseudo‐MRI avoids the intersection of head model surfaces which is a major challenge in multi‐layer volume conductor modeling for EEG. Although the results indicate its great potential and applicability in EEG, a separate validation would be required in the future to quantify the LE, address other challenges, and streamline the workflow for employing the pseudo‐MRI engine for EEG source imaging more confidently.

The software is intended to generate the pseudo‐MRI for MEG source imaging for subjects with normal brain anatomy. An ideal use case would be a group‐level research study with healthy adults. Pediatric MEG could also benefit from pseudo‐MRIs derived from a pediatric MRI template such as NIHPD (Fonov et al. 2011); however, a separate validation using such a template should be done to ensure its applicability and accuracy. The pseudo‐MRI engine could also be used for clinical populations with normal brain anatomy and when subcentimeter spatial accuracy is not mandatory.

## 5 Conclusion

The study shows that the suggested method provides a very close alternative to individual MRI for source imaging. Although an individual MRI would be mandatory for millimeter‐level spatial accuracy, pseudo‐MRI can be a viable alternative in MEG/EEG source imaging. The pseudo‐MRI can replace many expensive and time‐consuming steps such as MRI acquisition, data management, segmentation, and surface reconstruction; it can also streamline the whole analysis pipeline. Since an automated co‐registration is applied during the pseudo‐MRI generation, the work also replaces the need for a separate MEG‐MRI co‐registration. The pseudo‐MRI engine can be employed in various neuroscience research and clinical applications where subcentimeter precession is not mandatory. The method can also be utilized for real‐time source‐estimation workflows with greater accuracy than using, for example, the sphere model. The data‐dependent generation of pseudo‐MRI provides a less user‐dependent and more automated process to generate the pseudo‐MRI right after completing the digitization procedure.

## Supporting information


**Data S1.** Supporting Information: Figures.

**Table S1.** Time consumption for warping a template MRI (all surfaces and a voxel file, *T1.mgz*, of shape 256 × 256 × 256 voxels). The required time for warping increases, and the bending energy decreases with increasing number of control points (digitization points).
**Table S2.** Workstation specification and warping time taken by the pseudo‐MRI engine in a typical operation.

## Data Availability

The code for the *pseudo‐MRI engine* software used in this study is available at https://github.com/neurosignal/pseudo‐MRI‐engine along with a detailed tutorial for running the software. A brief tutorial is also provided at the end of the Supporting Information.
