# Supplementary material for: Pseudo‐MRI Engine for MRI‐Free Electromagnetic Source Imaging
Source: Hum Brain Mapp. 2025 Feb 4;46(2):e70148. doi: 10.1002/hbm.70148 (PMC11791934; doi:10.1002/hbm.70148)
Supplement: Supplementary file 1 — Data S1. Supporting Information: Figures. [file HBM-46-e70148-s001.pdf]

Supplementary material: Figures

Pseudo-MRI engine for MRI-free electromagnetic source imaging

[1,2,*]Amit Jaiswal, [2]Jukka Nenonen, [1,2]Lauri Parkkonen
[1]Department of Neuroscience and Biomedical Engineering, School of Science, Aalto University, Espoo, Finland.
[2]Megin Oy, Espoo, Finland.

*Corresponding author email: amit.jaiswal@aalto.fi

Short title: Pseudo-MRI engine for MEG/EEG source imaging.

1. Limitations of template MRI warping in Brainstorm with sparse and uneven scalp digitization

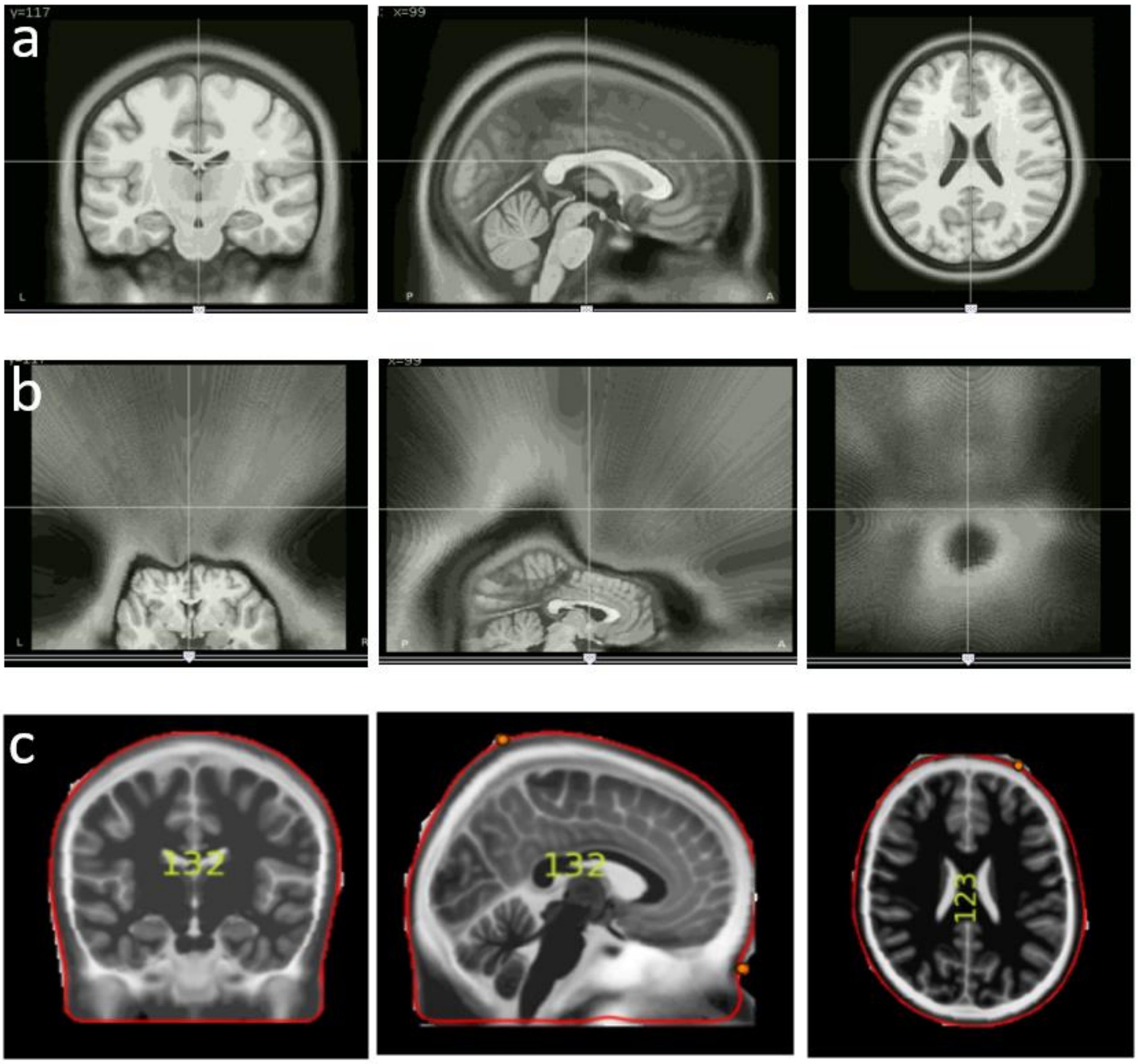

Fig. S1. Warping an MRI template employing Brainstorm and the pseudo-MRI engine for an adult subject with sparse and uneven scalp digitization— a) ICBM152 template MRI; warped template using b) Brainstorm software, and c) the pseudo-MRI engine. The total number of digitized scalp points was 26, and outliers were removed before warping the template MRI.

2. Comparison of Brainstorm-warping, MNE-Python scaling, and pseudo-MRI

The ICBM152 template MRI was warped/scaled for five subjects employing Brainstorm-warping, MNE-Python scaling, and the pseudo-MRI engine. These subjects were selected from the dataset described in Section 2.5. Each of the subjects had ~150 digitization points, and the warping/scaling was performed for five levels of digitization densities — 25, 50, 75, 100, and 150 points – by sub-sampling them from the existing digitization data. Points below the fiducials were removed before warping in Brainstorm, following 2% outlier rejection. In MNE-Python, using the co-registration GUI module, the template was scaled for all subjects using the five levels of digitization density, omitting points more than 5 mm from the scalp. In the pseudo-MRI engine, the warping was applied following several automated steps, such as removing points below a plane 5 mm lower to the fiducial points, checking the points distribution, and densifying them if the number remains < 50. Further, point (vertex) -wise distances of pseudo-MRIs inner-skull surfaces from that of the real MRI were computed for all subjects at each digitization level. Consequently, the same was done for the Brainstorm-warped MRIs and the scaled MRIs by MNE-Python. These computations were also repeated for scalp surface. The distance distributions, overlapping surfaces, and t-statistics are shown in Fig. S2a–c and S2d–f for the inner skull and scalp surface, respectively.

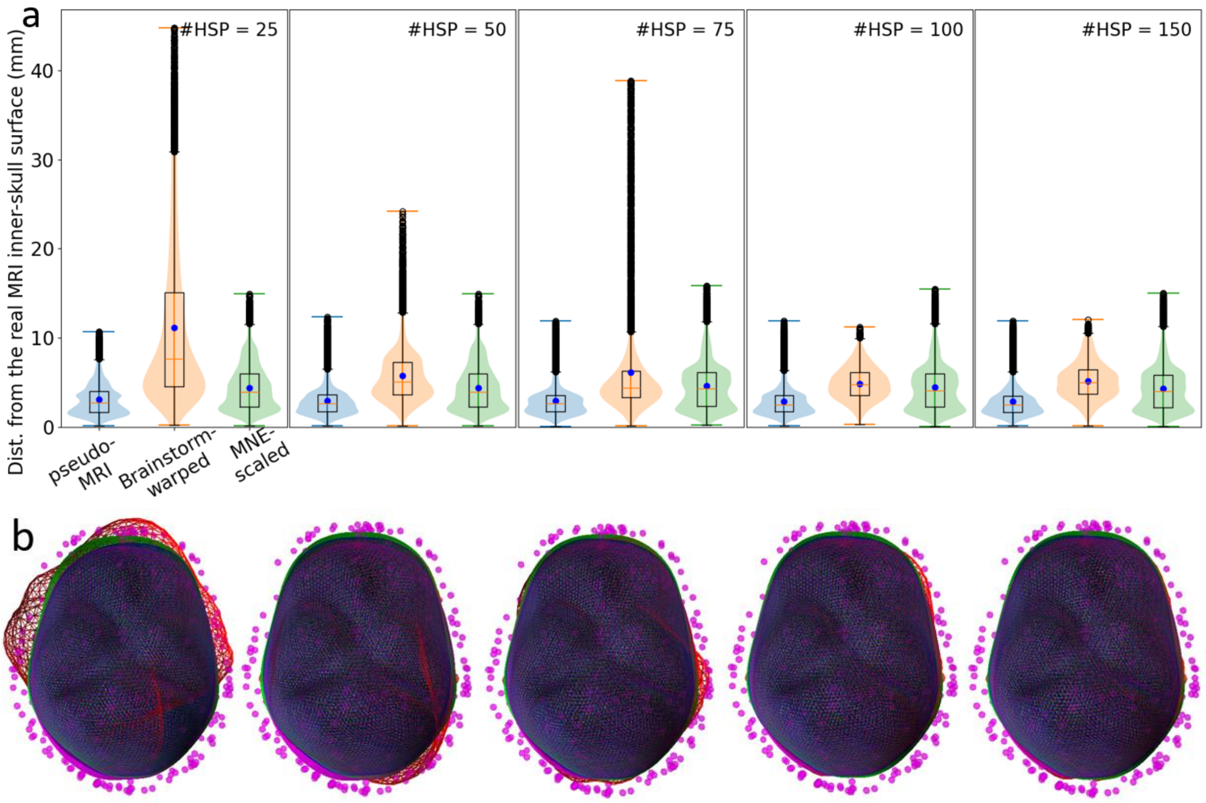

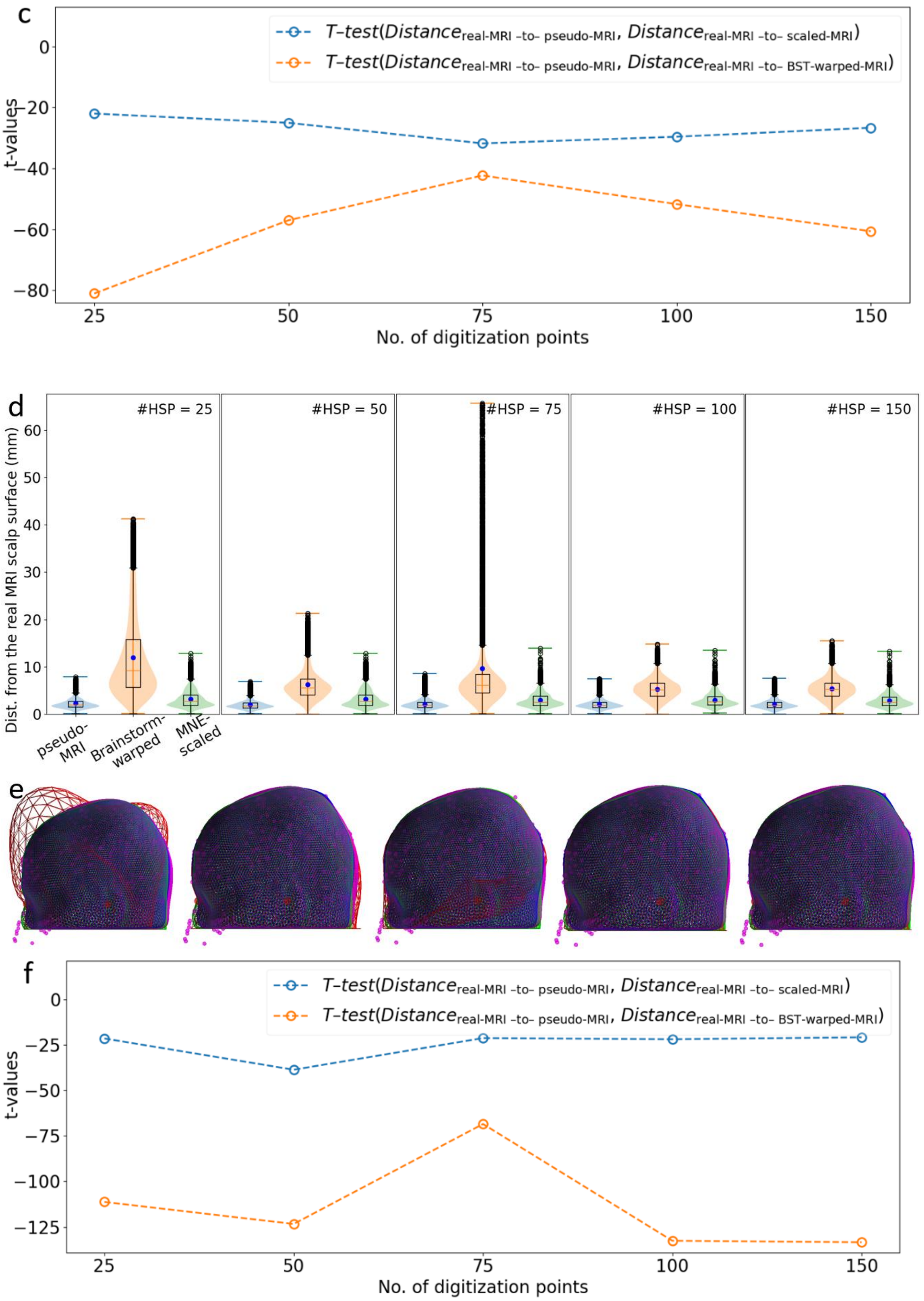

Fig. S2. For the inner-skull surfaces —a) distribution of vertex-wise surface distance from the real MRIs' inner-skull surfaces for the five subjects b) overlapping surfaces for a subject, c) *t*-values from the tests applied among surface distance distributions for pseudo-MRIs, Brainstorm (BST)-warped MRIs, and MNE-scaled MRIs. Further,

d), e), and f) represent the same for the scalp surfaces. In plots b and e, the real, pseudo, Brainstorm, and MNE-Python derived surfaces are shown in green, blue, red, and magenta, respectively; HSP stands for head shape (scalp) points.

3. Effect of deformity in template scalp surface

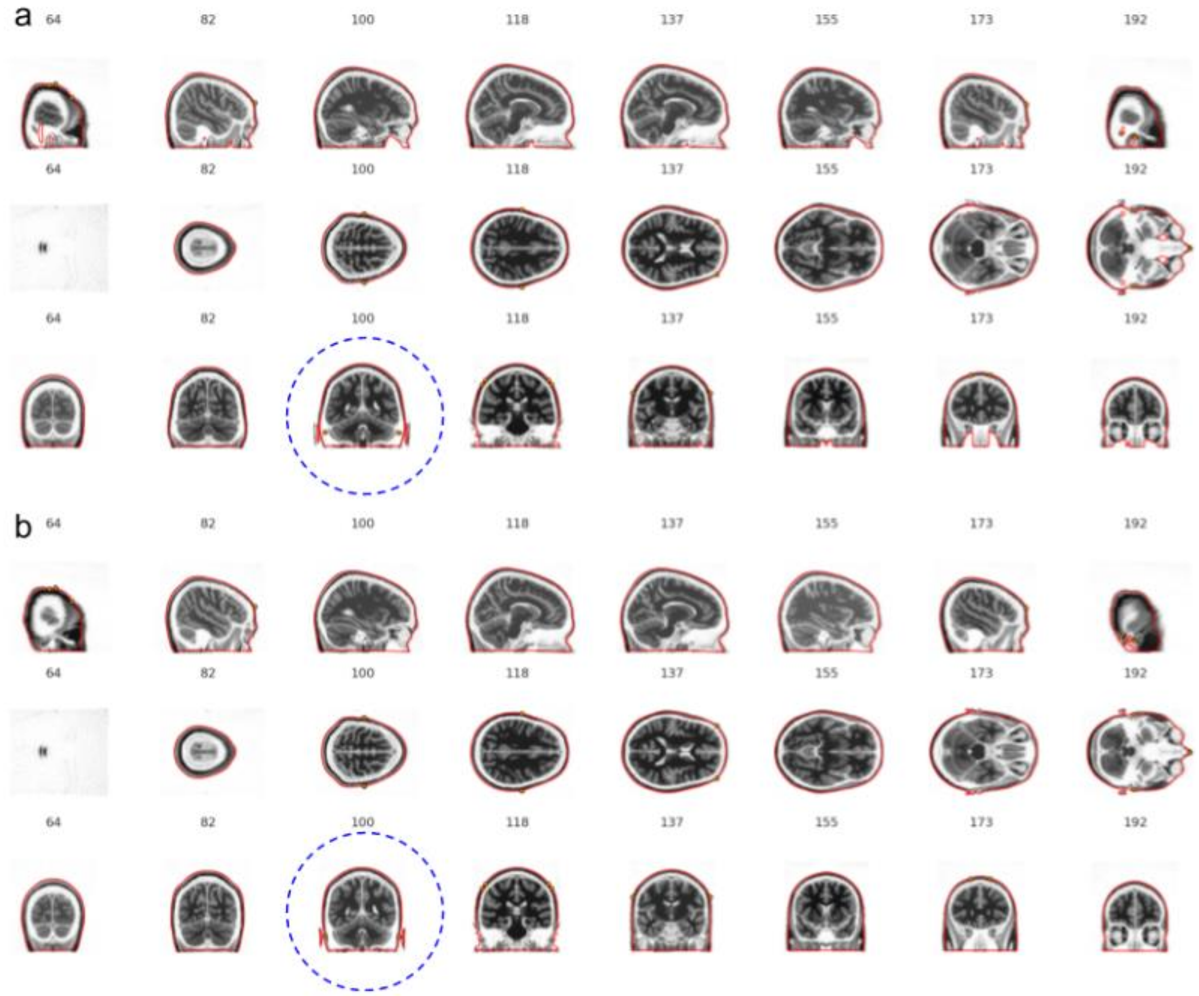

Fig. S3. Effect of deformity (reconstruction error) in the template (MNI152) scalp surfaces; pseudo-MRI view—a) when the warping transform was computed for the template scalp surface with reconstruction error (two cavities), b) when the template scalp surface was manually corrected by removing the cavities. In case (a), the cavities led to incorrect detection of control points on the template scalp surface that resulted in an inaccurate warping transform; see the improvement in the slices encircled by blue dotted lines.

4. Selection of fiducial points

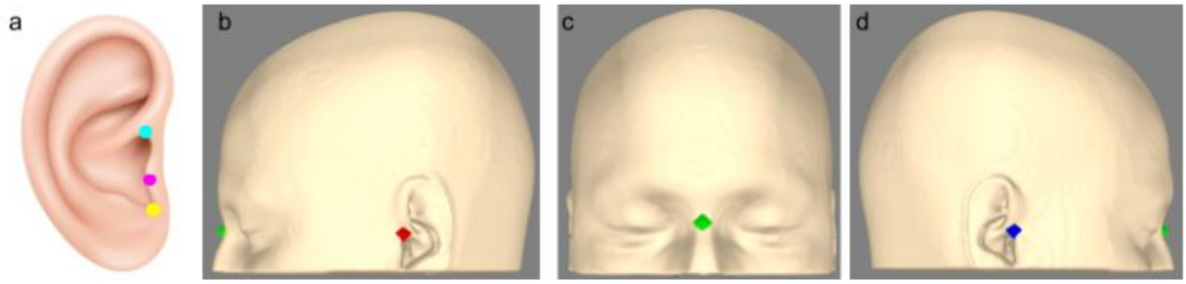

Fig. S4. Selection of fiducial points— a) *Helical crus and Targus intersection point* (cyan) is the most used location for LPA/RPA; however, the *Center of the tragus* (magenta) and *Intertragal notch* (yellow) are also used. The fiducial locations used in the study were b) LPA (red), c) Nasion (green), and d) RPA (blue).

5. User interfaces of the pseudo-MRI engine

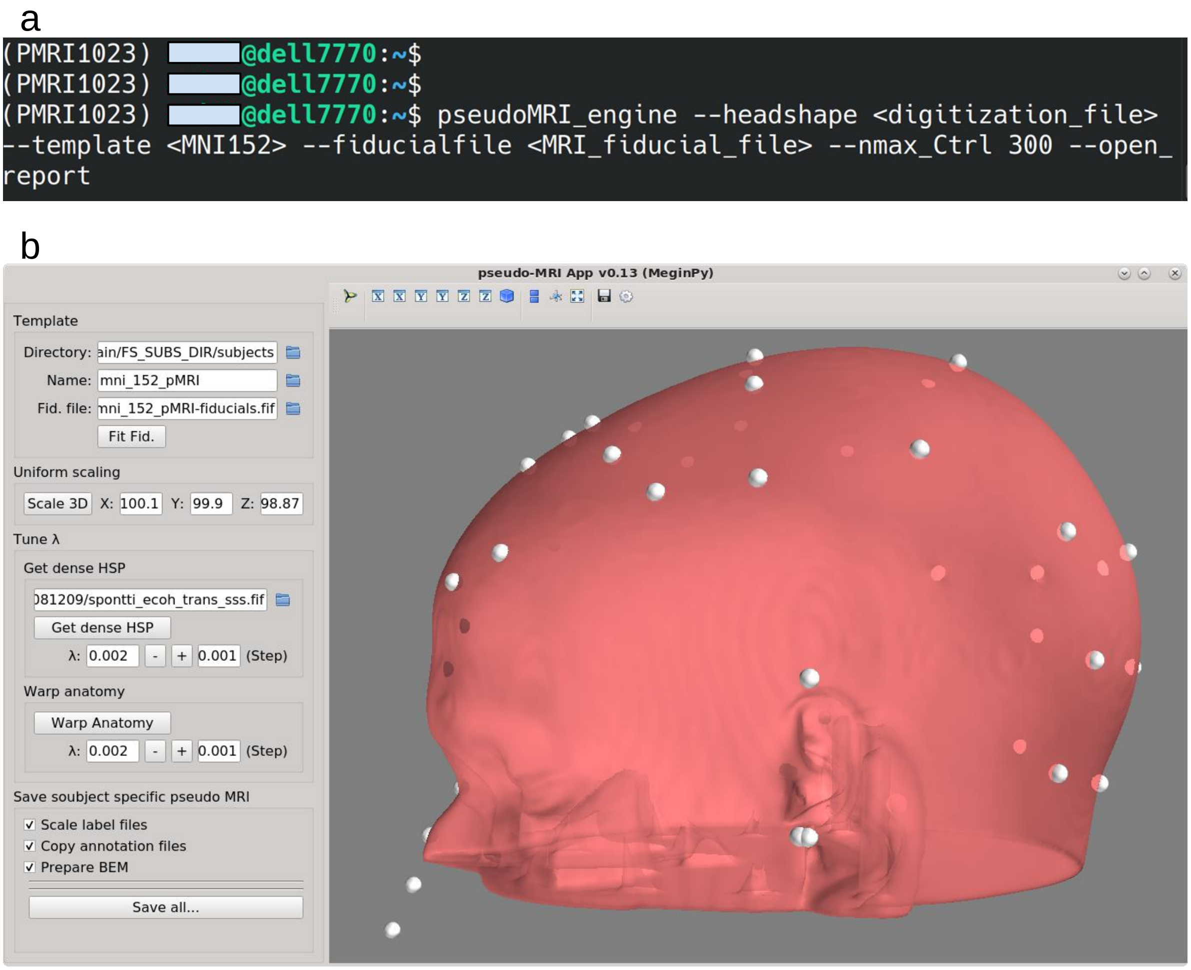

Fig. S5. a) The command line interface of the pseudo-MRI engine on a Linux machine, b) a view of the engine's graphical user interface.

6. Density and uniformity distribution of the digitization points across the test subject

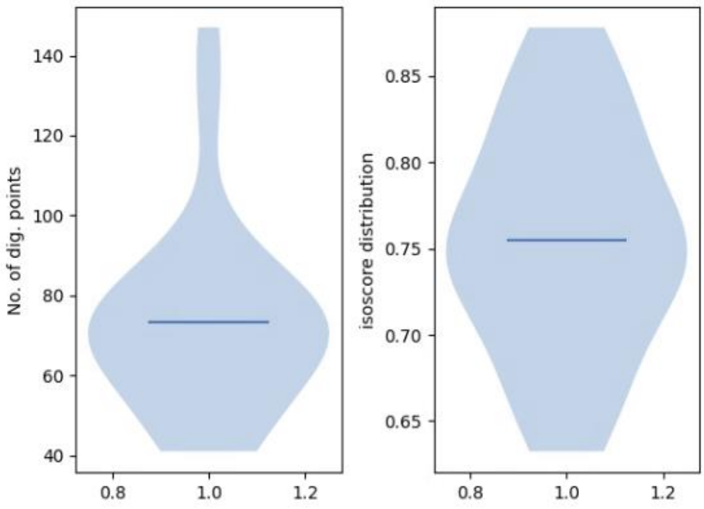

Fig. S6. Digitization points to density and uniformity across subjects.

7. Side length and triangle area distribution

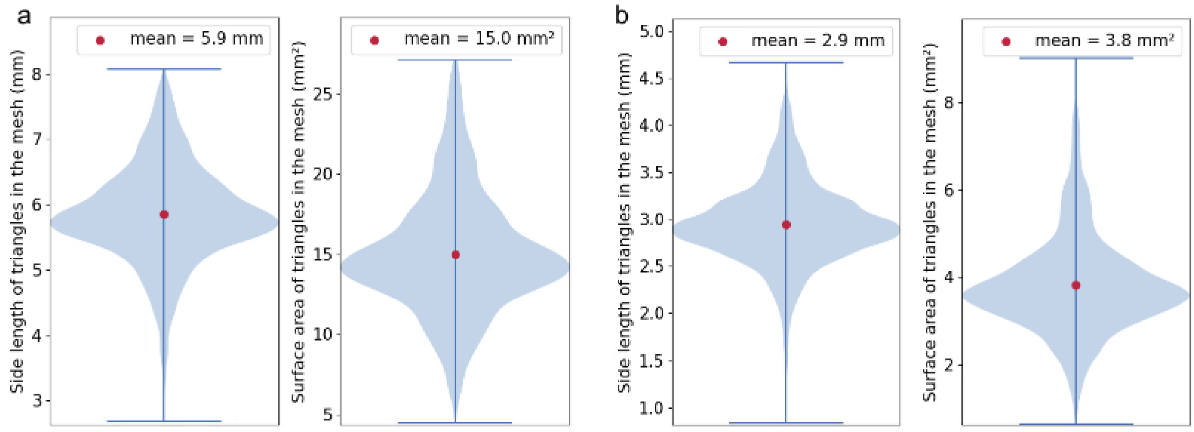

Fig. S7. Side length and triangle area distribution for the inner skull surface mesh with— a) ico4 subdivision, b) ico5 subdivision.

8. Hausdorff distance distribution for cortical surfaces

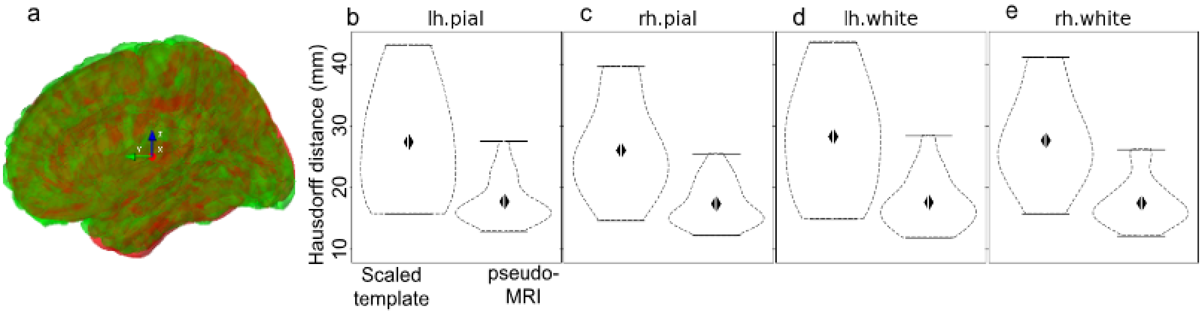

Fig. S8. a) Pial surfaces from real (green) and pseudo (red) MRI of a subject. Comparison of cortical surfaces from the scaled template and pseudo-MRI with that from the real MRI using the Hausdorff distance distribution for b) pial left surface, c) pial right surface, d) white left surface, and e) white right surface.

9. Correlation between depth of COMs of the brain regions for real and pseudo-MRIs

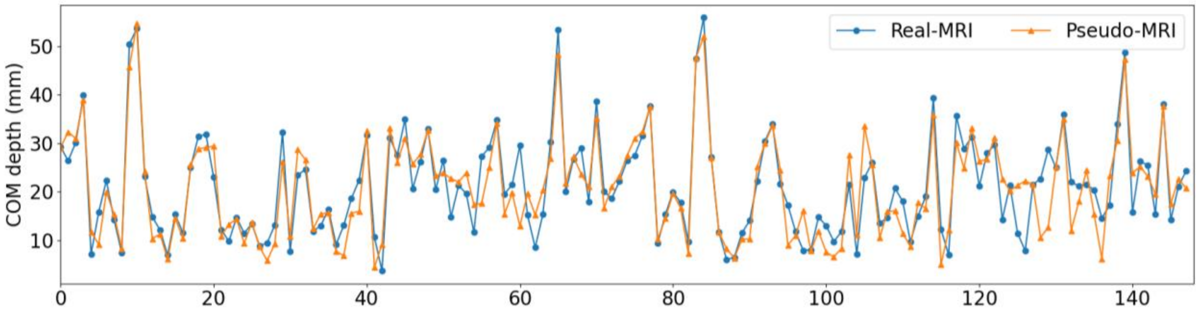

Fig. S9. Correlation between the depth of parcel's center-of-mass (COM) for the real and pseudo-MRIs, computed for the 148 brain regions as per the Destrieux atlas.

10. Correlations between parcel's surface area and volume for the real and pseudo-MRIs

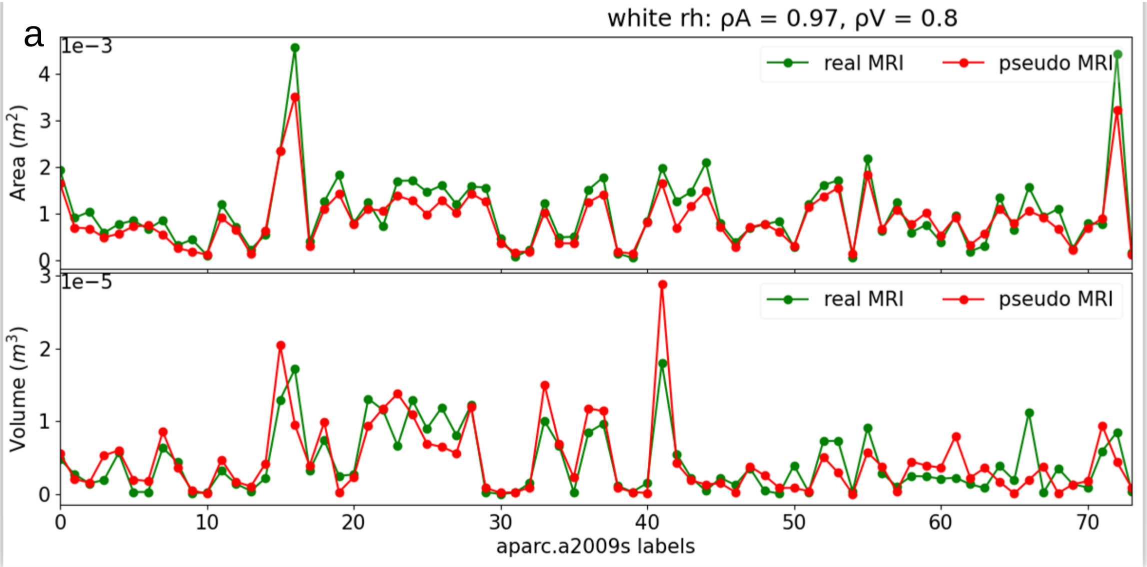

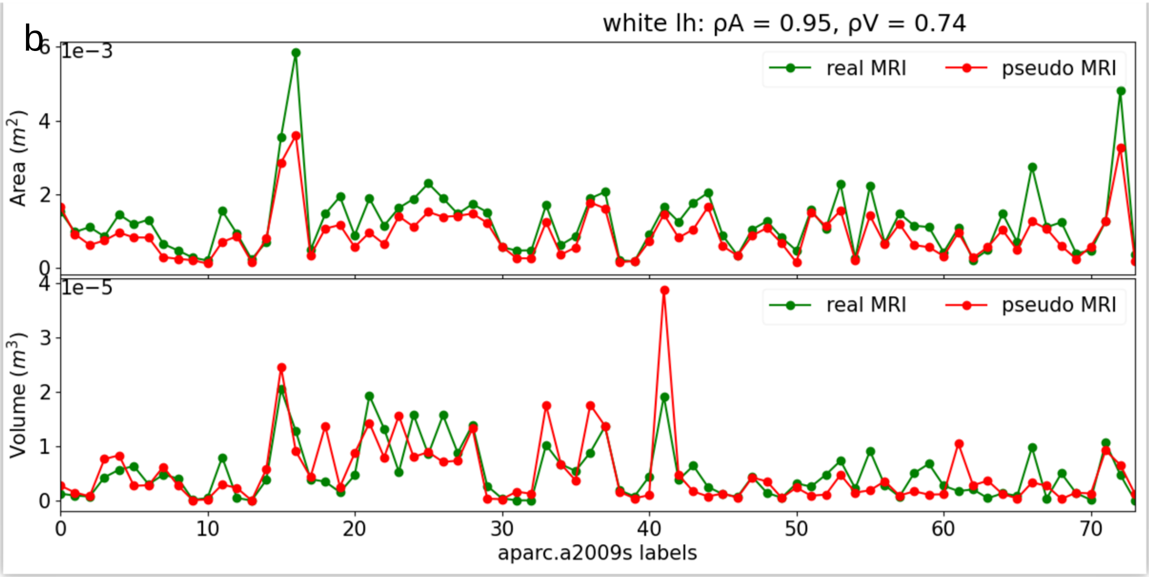

Fig. S10. a) Correlations between parcel's surface area and volume for the real and pseudo-MRIs' for the grey–white boundary surface in a) left and b) right hemispheres.

11. Distribution of dipole localization error for simulated data

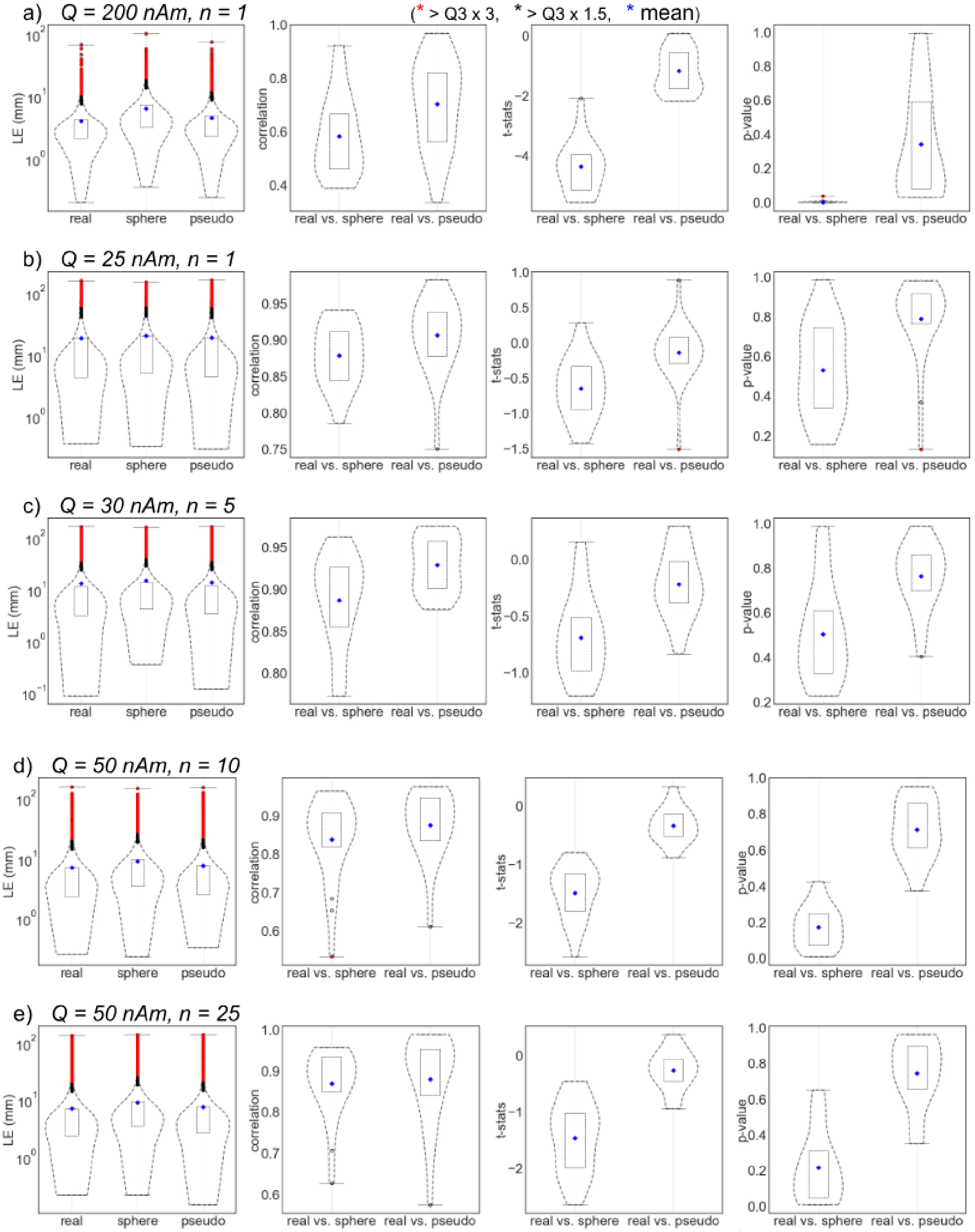

Fig. S11. From left to right on each row: the localization-error distribution, correlation, t-statistics, and p-values for simulated data for a) patches with one dipole at 200 nAm, b) patches with one dipole at 25 nAm, c) patches with five dipoles at 30 nAm, d) patches with ten dipoles at 50 nAm, and d) patches with 25 dipoles at 50 nAm.

12. A brief tutorial on pseudo-MRI engine software

Installation:

- Download the software:
  git clone https://github.com/neurosignal/pseudo-MRI-engine.git
- Change the current direct to pseudo-MRI-engine
  cd pseudo-MRI-engine
- Set up a Python environment, for example as:
  conda activate < your environment>
- Run to check and install all dependencies for the pseudo-MRI engine:
  pip install -r requirements.txt
- Check the installation:
  python pseudoMRI_engine.py –help

-p PSEUDO_MRI_NAME, --pseudo_MRI_name PSEUDO_MRI_NAME
subject name
-pd PSEUDO_MRI_DIR, --pseudo_MRI_dir PSEUDO_MRI_DIR
Parent directory for the pseudo-MRI folder (optional)
-dig HEADSHAPE, --headshape HEADSHAPE
File with headshape digitization information
-t TEMPLATE_MRI_NAME, --template_MRI_name TEMPLATE_MRI_NAME
Template MRI name
-td TEMPLATE_MRI_DIR, --template_MRI_dir TEMPLATE_MRI_DIR
Parent directory of the template MRI folder
-fids FIDUCIAL_FILE, --fiducial_file FIDUCIAL_FILE
Fiducial file of the template MRI
-paloc PREAURI_LOC, --preauri_loc PREAURI_LOC
LPA/RPA location considered during the head digitization; choose from CrusHelix, Targus, and ITnotch.
-nctrl NMAX_CTRL, --nmax_Ctrl NMAX_CTRL
Number of maximum control points.
-densify, --dense_hsp
densify HSP?
-v, --verbose *verbose mode or not?*
-o, --open_report *open report or not when completed?*

Preparation of a template MRI:

Segment a template MRI, for example MNE152, using *recon-all routine* of FreeSurfer (or FastSurfer). Also compute head model and scalp surfaces using MNE-Python routines. Furthermore, the fiducial points for the template MRI can be identified using MNE-Python coregistration module and saved as FIFF file with an appropriate pattern from CrusHelix, Targus, and ITnotch.

Running the software

python pseudoMRI_engine.py --pseudo_MRI_name <subject ID> --pseudo_MRI_dir <pseudo-MRI folder> --headshape <headshape file> --template_MRI_name <name of template MRI folder> --

template_MRI_dir <the parent directory of the template MRI folder> --fiducial_file <fiducial file of the template MRI> --preauri_loc <the position of the LPA/RPA considered during the head digitization> --nmax_Ctrl <maximum number of the control points to compute warping> --dense_hsp <set this flag to force densifying the digitization points if too sparse> --open_report <set this flag to open the HTML report file in the end>
