# Supplementary material for: Pseudo‐MRI Engine for MRI‐Free Electromagnetic Source Imaging
Source: Hum Brain Mapp. 2025 Feb 4;46(2):e70148. doi: 10.1002/hbm.70148 (PMC11791934; doi:10.1002/hbm.70148)
Supplement: Supplementary file 2 — Table S1. Time consumption for warping a template MRI (all surfaces and a voxel file, T1.mgz, of shape 256 × 256 × 256 voxels). The required time for warping increases, and the bending energy decreases with increasing number of control points (digitization points). Table S2. Workstation specification and warping time taken by the pseudo‐MRI engine in a typical operation. [file HBM-46-e70148-s002.docx]

**Supplementary materials: Tables**

**Pseudo-MRI engine for MRI-free electromagnetic source imaging**

^1,2,*^Amit Jaiswal, ^2^Jukka Nenonen, ^1,2^Lauri Parkkonen

^1^Department of Neuroscience and Biomedical Engineering, School of Science, Aalto University, Espoo, Finland.

^2^Megin Oy, Espoo, Finland.

*Corresponding author email: [amit.jaiswal@aalto.fi](mailto:amit.jaiswal@megin.fi)

*Short title:* Pseudo-MRI engine for MEG/EEG source imaging.

# **1. Time consumption for warping**

| No. of control points | Duration  (hh:mm:ss:ms) | Bending energy | Points to surface mean distance (mm)  (before - > after) |
| --- | --- | --- | --- |
| 5 | 00:01:40.80 | -.000017 | 6.5 - >1.9 |
| 10 | 00:01:49.63 | -0.00139 | 7.0 - > 1.5 |
| 15 | 00:01:42.63 | -0.00262 | 7.1 - > 1.3 |
| 25 | 00:01:54.64 | -0.00590 | 7.8 - > 1.2 |
| 40 | 00:02:01.25 | -0.00836 | 8.0 - > 1.1 |
| 65 | 00:02:31.55 | -0.01666 | 8.0 - > 1.1 |
| 105 | 00:03:15.21 | -0.02439 | 8.0 - > 1.0 |
| 170 | 00:04:03.47 | -0.04269 | 8.0 - > 0.9 |
| 275 | 0:04:59.66 | -0.06868 | 8.0 - > 0.9 |
| 445 | 00:07:42.32 | -0.13293 | 8.0 - > 0.8 |

Table. S1. Time consumption for warping a template MRI (all surfaces and a voxel file, *T1.mgz,* of shape 256x256x256 voxels). The required time for warping increases, and the bending energy decreases with increasing number of control points (digitization points).

# **2. Time consumption and a workstation specification used in the study**

| Workstation specification | Data details and time consumption for warping |
| --- | --- |
| Operating System: Kubuntu 20.04  KDE Plasma Version: 5.18.8  KDE Frameworks Version: 5.68.0  Qt Version: 5.12.8  Kernel Version: 5.15.0-56-generic  OS Type: 64-bit  Processors: 24 × 12th Gen Intel® Core™ i7-12850HX  Memory: 62.5 GiB | No. of control points = 100  Rectification of control points = 2 s  Computation of warping coefficients = 10 s  Warping of head surfaces = 5 s  Warping of cortical meshes = 6 s  Warping of atlas labels = 4 s  Warping of voxel files with 256x256x256 voxel = ~100s/file |

Table S2. Workstation specification and warping time taken by the pseudo-MRI engine in a typical operation.
